# Expert Consensus Survey on Digital Health Tools for Patients With Serious Mental Illness: Optimizing for User Characteristics and User Support

**DOI:** 10.2196/mental.9777

**Published:** 2018-06-12

**Authors:** Ainslie Hatch, Julia E Hoffman, Ruth Ross, John P Docherty

**Affiliations:** ^1^ Otsuka America Pharmaceutical, Inc Princeton, NJ United States; ^2^ Behavior Dx San Jose, CA United States; ^3^ Ross Editorial Port Townsend, WA United States

**Keywords:** biomedical technology, patient engagement, severe mental disorders

## Abstract

**Background:**

Digital technology is increasingly being used to enhance health care in various areas of medicine. In the area of serious mental illness, it is important to understand the special characteristics of target users that may influence motivation and competence to use digital health tools, as well as the resources and training necessary for these patients to facilitate the use of this technology.

**Objective:**

The aim of this study was to conduct a quantitative expert consensus survey to identify key characteristics of target users (patients and health care professionals), barriers and facilitators for appropriate use, and resources needed to optimize the use of digital health tools in patients with serious mental illness.

**Methods:**

A panel of 40 experts in digital behavioral health who met the participation criteria completed a 19-question survey, rating predefined responses on a 9-point Likert scale. Consensus was determined using a chi-square test of score distributions across three ranges (1-3, 4-6, 7-9). Categorical ratings of first, second, or third line were designated based on the lowest category into which the CI of the mean ratings fell, with a boundary >6.5 for first line. Here, we report experts’ responses to nine questions (265 options) that focused on (1) user characteristics that would promote or hinder the use of digital health tools, (2) potential benefits or motivators and barriers or unintended consequences of digital health tool use, and (3) support and training for patients and health care professionals.

**Results:**

Among patient characteristics most likely to promote use of digital health tools, experts endorsed interest in using state-of-the-art technology, availability of necessary resources, good occupational functioning, and perception of the tool as beneficial. Certain disease-associated signs and symptoms (eg, more severe symptoms, substance abuse problems, and a chaotic living situation) were considered likely to make it difficult for patients to use digital health tools. Enthusiasm among health care professionals for digital health tools and availability of staff and equipment to support their use were identified as variables to enable health care professionals to successfully incorporate digital health tools into their practices. The experts identified a number of potential benefits of and barriers to use of digital health tools by patients and health care professionals. Experts agreed that both health care professionals and patients would need to be trained in the use of these new technologies.

**Conclusions:**

These results provide guidance to the mental health field on how to optimize the development and deployment of digital health tools for patients with serious mental illness.

## Introduction

### The Potential of Digital Health Tools for Psychiatric Practice

The provision of acute and ongoing mental health care around the world continues to face challenges ranging from cost, access, and scalability to stigma-related concerns resulting in insufficient and inefficient treatment for many individuals with serious mental illness (SMI) [1]. Digital health is a rapidly advancing field that presents opportunities to significantly enhance health and health care. Digital health encompasses categories such as mobile health (mHealth), health information technology, wearable devices, telehealth or telemedicine, and personalized medicine [2]. Digital health tools have the potential to empower patients, improve access to care, enhance communication between patients and providers, improve health outcomes, and make health care processes and decisions more efficient and cost-effective [2-5]. Digital health tools are increasingly being adopted for use independently or in the context of traditional care and have been effective for monitoring and improving physical health in patients with a variety of conditions such as diabetes [6-8]. Interest in applying digital technologies to psychiatric practice has been increasing since the early 2000s, and recommendations for future research in this area were issued in 2013 by a technical expert panel convened by the Agency for Healthcare Research and Quality and the National Institute of Mental Health [9-11]. Examples of interventions that have used digital health tools in patients with SMI include mobile-based assessments and interventions [12-15], computerized psychotherapies [16], cognitive remediation [17,18], family psychoeducation interventions [19], and interventions specifically targeting medication adherence [20].

Although digital health tools hold significant promise for the improvement of various elements of behavioral health intervention, and surveys have indicated that people with SMI are enthusiastic about using technology [21,22], adoption has been slow, testing of outcomes from acceptability to effectiveness has been limited, and codified development and deployment strategies have been lacking [23-30]. These preliminary efforts need to be undertaken before their effectiveness can be tested and verified so that the resulting findings can be incorporated into the redesign and reconfiguration of digital health tools to provide an optimal solution.

Moreover, it is clear that advances in the digital health tool field depend on the identification of the right patients, providers, and settings or conditions to maximize clinical effects and minimize risks. As the field is emerging, these considerations are yet to be fully studied and elucidated. However, practical guidance to facilitate the selection and use of these tools, which must include empirically derived guidance on appropriate user selection and clinical settings, as well as education and resource requirements for patients, may encourage adoption among all target users. In addition, consideration of implementation strategies and education about unintended consequences and known risks associated with the use of digital health tools, as well as mitigation strategies for each, will also be important.

**Figure 1:**
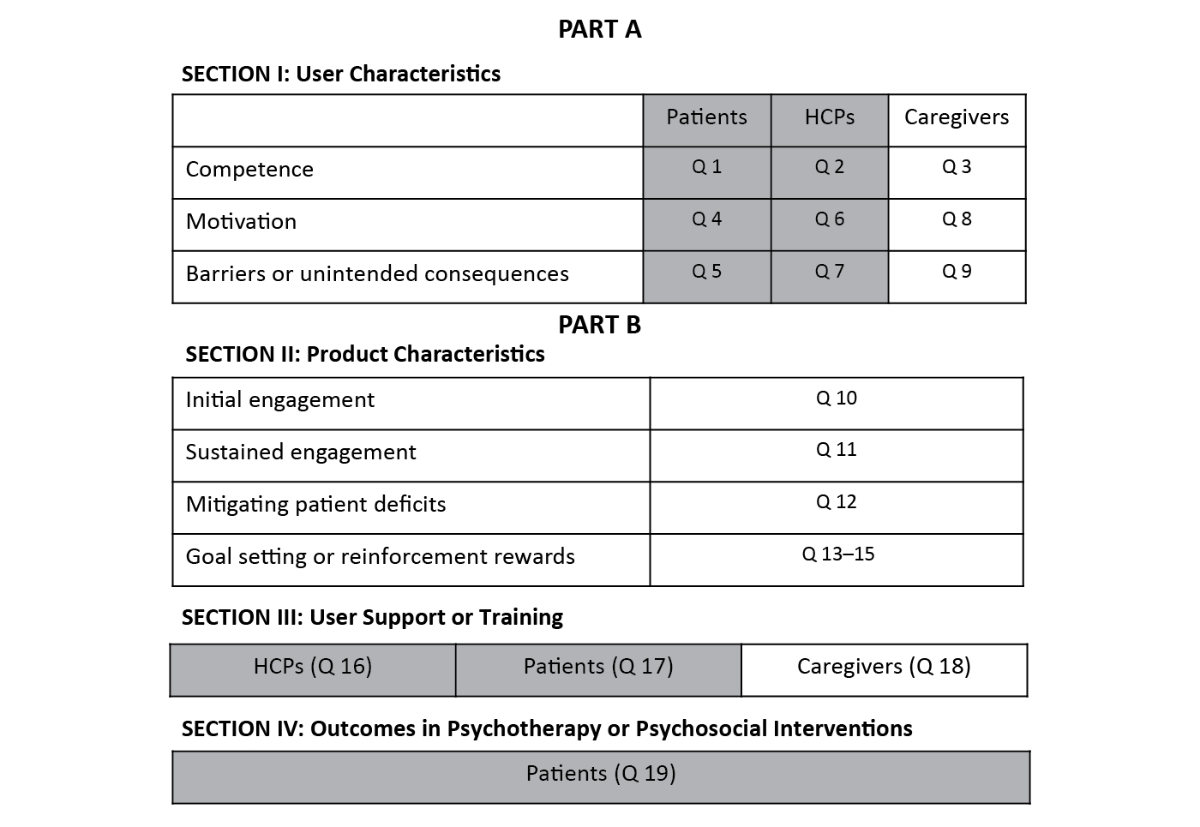
Survey structure. Results from highlighted sections and questions are reported in this paper. HCP: health care professional.

Ideally, these considerations will be defined with empirical data; however, until these data emerge, academic clinicians with expertise in the field of behavioral health technology can provide valuable feedback on these issues. Results from the survey described here can be used to develop recommendations for physicians and other allied health care professionals (HCPs; eg, psychologists, case managers, and social workers) concerning the use of digital health tools in clinical practice.

### Rationale for the Use of the Expert Consensus Methodology

The expert consensus survey methodology was developed as a means of providing quantitative and reliable data on which to base best practice recommendations for clinical areas that are not well covered by definitive research [31]. Since 1996, this method has been applied to 26 areas of clinical practice, including schizophrenia, bipolar disorder, depressive disorders, attention-deficit hyperactivity disorder, obsessive-compulsive disorder, and dementia, with data presented in more than 75 publications since 1996 [32-34]. Given the limited empirical research concerning the use of digital health tools in psychiatry, the expert consensus survey methodology was used to develop recommendations for this emerging field.

The overall goals of this expert consensus survey were to assess expert opinion on target user characteristics, product features, and user support (training or resources) to facilitate adoption of and successful engagement with digital health tools by patients with SMI (Figure 1). This report describes results from subsections of the survey that focused on user characteristics (patients and HCPs) and user support. Results from this survey can provide guidance to optimize the development and use of digital health tools in clinical psychiatry.

## Methods

### Expert Panel

Experts were identified based on authorship on published articles and congress abstracts and the following criteria: work involving the design, development, validation, optimization, evaluation, implementation, and dissemination of assessment or technology-assisted treatment interventions in psychiatry. Of the 82 experts invited to participate in the survey via email, 17 did not respond, and 13 declined to participate. Of the remaining 52 experts who received the survey, 40 (77%) completed part A of the survey, and 39 (75%) completed parts A and B. This study was exempt from review by an institutional review board; it involved only the use of survey procedures, did not involve children, and the data were anonymized such that responses could not be linked to an individual respondent.

The respondents had an average of 16 years of experience in clinical practice, 20.5 years in clinical research, and 5 years in basic research. They reported currently spending approximately 15% of their professional time on average in clinical work, with 28% (11/40) spending 25% of more of their time in clinical work and 73% (29/40) spending less than 25% of their time. Of the 40 respondents, 63% (25/40), 45% (18/40), and 60% (24/40) reported extensive experience working with patients with schizophrenia, bipolar disorder, and major depressive disorder (MDD), respectively; 55% (22/40), 38% (15/40), and 55% (22/40) had experience with developing or implementing digital health tools in patients with schizophrenia, bipolar disorder, and MDD, respectively. Most of the experts worked in an academic clinical or research setting, although some also worked in the public sector or in private practice. All the 40 experts had participated in a research project using mHealth or behavioral assessment tools, ambulatory monitoring, ecological momentary assessment, or experience sampling techniques (30/40, 75%); had participated in a research project using mobile mental health treatment tools or ecological momentary intervention techniques (25/40, 63%); or had prescribed or recommended a computerized treatment or mobile app to a patient (26/40, 65%). Moreover, 33% (13/40) and 38% (15/40) of experts had performed all three or two of the above, respectively. The expert panel had the most experience with mobile apps, computerized treatment programs, and websites.

### Creating the Survey

On the basis of their experience, as well as review of current literature in the field of digital health tools, the authors participated in multiple iterative group discussions to arrive at the survey structure and questions. This survey used the expert consensus methodology [31]. The developers created a 19-question survey with 540 options using Adobe Forms software (Adobe Systems Incorporated, San Jose, CA). The survey was divided into two parts (A and B) and took participants approximately 2 hours to complete either online or as a PDF document. The respondents were paid a fee for participating. In responding to the survey, the experts were instructed to assume that the patient has schizophrenia, bipolar disorder, or MDD. The term *“health care professional”* was used to refer to any professional who provides health care services to patients with ≥1 of these three disorders (eg, psychiatrists, psychologists, nurses, clinical social workers, or case managers). Although the survey covered a number of issues related to the use of digital health tools in patients with psychiatric disorders (Figure 1), this report focuses on questions regarding patients and HCPs that covered the following three main areas: (1) characteristics of the most appropriate users of digital health tools related to their ability to engage with and use a digital health tool (target user *competence*: questions 1, 2, and 19), (2) potential benefits of and barriers to use of digital health tools (target user *motivation*: questions 4-7), and (3) training resources needed to optimize use of digital health tools (training resources: questions 16 and 17). Graphic and tabular results for these 9 questions are presented in Multimedia Appendix 1. The experts were asked to rate the options based on their experiences using the technology with which they were most familiar in patients with psychiatric illness in clinical research and practice settings and based on their best understanding of the current literature.

The experts rated 529 of the survey options using a rating scale slightly modified from a format developed by the RAND Corporation [35]. The scale ranged from 1 to 9 (eg, 1=not at all likely to motivate and 9=extremely likely to motivate, or 1=not important at all to 9=extremely important; to avoid confusion, for all questions rated with the 9-point scale, a score of 9 was used to indicate the most positive options and a score of 1 the most negative options. For example, as shown in Multimedia Appendix 1, in question 1, a score of 9 was used to indicate patient characteristics that were extremely likely to promote engagement and ability to use a digital health tool, whereas in question 5, a score of 1 was used to indicate a characteristic that had significant potential to be a barrier or an unintended consequence when using a digital health tool). For the other 11 options, respondents were asked to write in a response.

### Data Analysis

For each option scored on the rating scale, the presence of consensus was defined as a distribution unlikely to occur by chance by performing a chi-square test (*P*=.05) of the distribution of scores across the three ranges of scores (1-3, 4-6, 7-9). The mean and 95% CI were calculated for the ratings. Categorical ratings of first, second, or third line were designated based on the lowest category in which the CI fell, with a boundary >6.5 for first line and 3.5 to 6.5 for second-line options. Although significance of differences among options was not statistically evaluated, if the CIs do not overlap, that generally indicates a significant difference between options by *t* test, with a wider gap indicating a more significant difference (ie, a smaller *P* value).

## Results

### Graphic and Tabular Results

**Figure 2:**
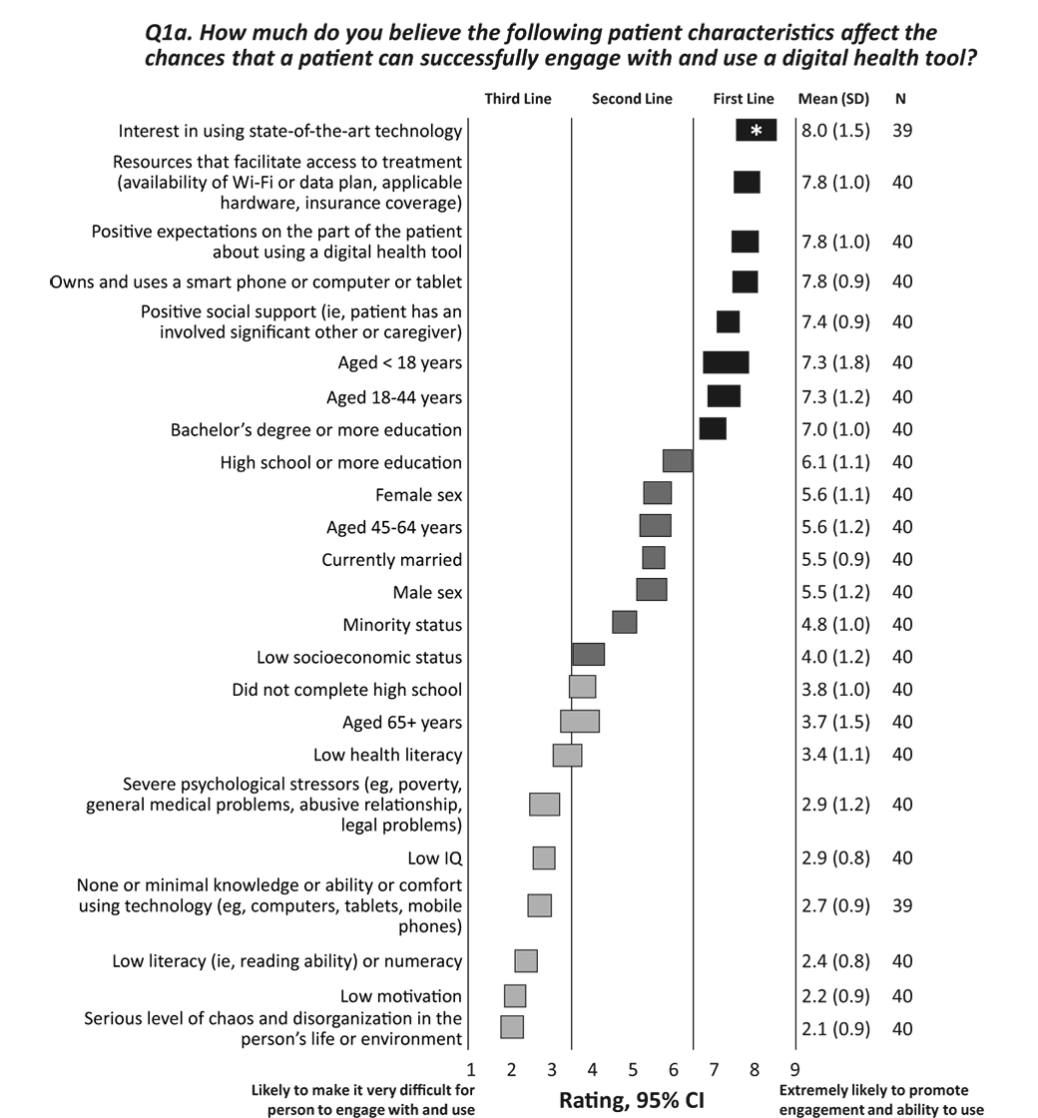
Patient characteristics that affect engagement with and use of a digital health tool. The CIs for each option are shown as horizontal bars; consensus is indicated by a shaded bar; the number of respondents, the mean rating, and SD are given in the column on the right. Note that some respondents did not rate some options; therefore, the number of respondents is 39 or 40 for the options in this survey question. The experts rated each item on a scale of 1 to 9, with 1=likely to make it very difficult for person to engage with and use, 5=not likely to influence the ability to engage with or use, and 9=extremely likely to promote engagement and ability to use. *Highest rating of 9 given by ≥50% of experts.

Graphic and tabular results of responses for all 9 questions are presented in Multimedia Appendices 1 and 2, respectively. For an explanation of how to interpret the graphic results, refer to Figure 2. Options on which consensus was not achieved are shown in the graphic results by an unshaded box and do not represent experts’ consensus recommendations. The tabular results show the full range of responses to these questions.

### Patients

#### Characteristics of Patients Likely to Influence Ability to Use Digital Health Tools

To optimize the use of digital health tools in patients with SMI, it is essential to identify patients who are most likely to adopt digital technologies. The experts were asked to rate how certain characteristics affect patients’ ability to successfully engage with and use a digital health tool. The responses are shown in Figure 2 (and question 1a in Multimedia Appendix 1). A patient’s interest in using state-of-the-art technology received a first-line rating from 90% (35/39) of experts, followed by access to necessary resources (eg, Wi-Fi and hardware), positive expectations about use of the digital health tool, ownership and use of a smartphone or computer or tablet, and positive social support. Patients with a serious level of chaos or disorganization in their lives, low literacy, or low motivation were considered the least likely candidates to use digital health tools.

**Figure 3:**
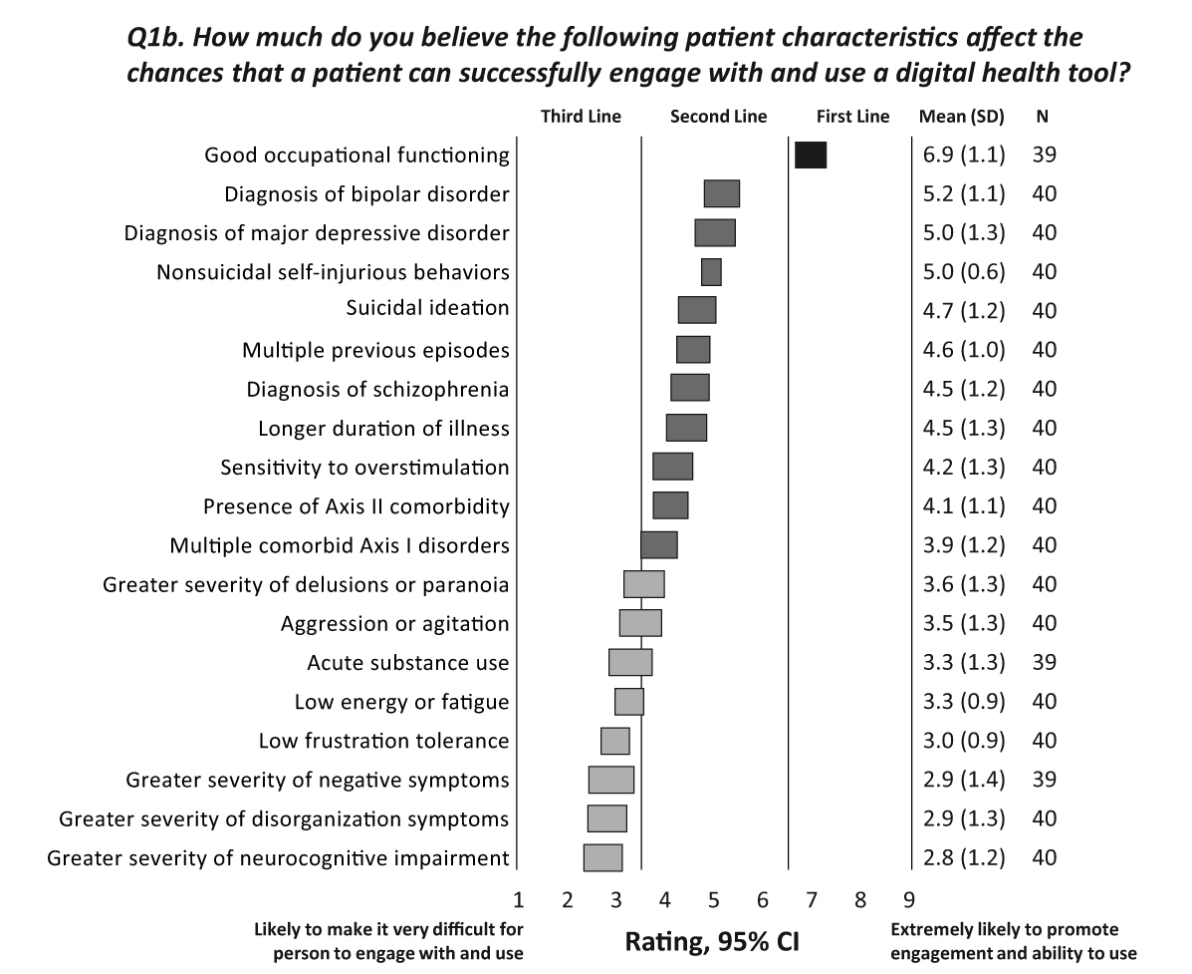
Disease-related characteristics that affect a patient’s ability to engage with and use a digital health tool. The CIs for each option are shown as horizontal bars; consensus is indicated by a shaded bar; the number of respondents, the mean rating, and SD are given in the column on the right. Note that some respondents did not rate some options; therefore, the number of respondents is 39 or 40 for the options in this survey question. The experts rated each item on a scale of 1 to 9, with 1=likely to make it very difficult for person to engage with and use, 5=not likely to influence the ability to engage with or use, and 9=extremely likely to promote engagement and ability to use.

The experts were next asked about the extent to which patients’ diagnoses and the signs and symptoms of their illness are likely to influence their ability to successfully engage with and use digital health tools. The responses are summarized in Figure 3 (and question 1b in Multimedia Appendix 1). Good occupational functioning was the only option rated first line (ie, most likely to promote the use of digital health tools). Although diagnoses of schizophrenia, bipolar disorder, or MDD were not independently considered likely to influence the ability to use a digital health tool, a number of symptomatic presentations associated with these disorders were rated as likely to make it difficult for a person to engage with a digital health tool. These included more severe positive, negative, disorganized, and neurocognitive symptoms; acute substance abuse; agitation or aggression; and low energy or frustration or tolerance.

Among the appraisals and experiences likely to affect patients’ ability to engage with and use a digital health tool (see question 1c in Multimedia Appendix 1), all respondents considered a perception by patients that the digital health tool is beneficial as most likely to promote successful engagement and ability to use the digital health tool (mean rating 8.2, SD 0.7). Other characteristics highly likely to promote patient use of digital health tools included the following: agreement with tasks and goals of digital treatment (mean 7.9, SD 0.8), self-efficacy beliefs about being able to use the device (mean 7.9, SD 0.9), a good therapeutic alliance (mean 7.7, SD 0.9), willingness to complete tasks or homework between treatment sessions (mean 7.7, SD 0.9), a history of good treatment adherence (mean 7.4, SD 0.9), and readiness to change (mean 7.3, SD 1.1). Negative past experience with treatment and limited insight into their illness were rated as likely to make it more difficult for patients to engage with or use digital health tools.

To assess how patient and disease characteristics affecting the use of digital health tools compare with those that affect psychotherapy or psychosocial interventions in general, the experts were asked to rate how the same patient characteristics (except those related to technology) affect the chances of achieving favorable outcomes in any psychotherapy or psychosocial intervention (see question 19 in Multimedia Appendix 1). The responses of the experts were similar to those obtained when they were asked about characteristics that would affect use of digital health tools. They considered that the patients who were most likely to achieve favorable outcomes were those patients who had positive expectations about the therapy (mean 7.8, SD 1.0), resources that facilitate access to treatment (mean 7.7, SD 0.9), positive social support (mean 7.5, SD 1.4), and good occupational functioning (mean 7.1, SD 0.8). Sex, age, marital status, socioeconomic status, and high school educational level were considered unlikely to influence outcomes (all received second-line ratings). Low motivation, a serious level of chaos or disorganization in patients’ lives or environment, severe psychosocial stressors (eg, poverty and general medical problems), substance abuse problems, greater severity of symptoms, and greater severity of neurocognitive impairment were considered likely to have an adverse effect on outcomes.

#### Potential Benefits and Barriers or Unintended Consequences for Patients

The experts were told to assume that certain benefits had been demonstrated to be benefits for digital health tools for SMI patients. They were then asked how likely these various potential benefits would be to motivate patients to use digital health tools (see question 4 in Multimedia Appendix 1). Table 1 lists benefits that were rated as highly likely to motivate patients (options rated first line that received a mean rating ≥7.0). The following two options were rated high second line and received mean ratings ≥7.0: (1) reduction in the number of hospitalizations and (2) increased social engagement enabled by technology. There was no consensus among experts on the potential of digital health tools to reduce health care costs as a motivating factor for patients.

The experts were asked to consider the average patient with SMI and rate how likely it was that various items would be a potential barrier or lead to unintended consequences for patients using digital health tools (see question 5 in Multimedia Appendix 1). The items rated by the experts as having a substantial potential to be a barrier or unintended consequence of patients’ use of a digital health tool (third-line options with mean ratings ≤3.5) are listed in Table 1. No consensus was achieved concerning the following as potential barriers: increase in family conflict because of information provided by the digital health tool, depersonalization of patient care and potential damage to therapeutic relationship, patient’s use of the digital health tool in isolation leading to decreased face-to-face interaction with clinician or treatment team, and patient being disappointed by not receiving prompt response from the HCP. In addition, no consensus was achieved concerning the following two items specific to patients with psychosis: (1) possible exacerbation of paranoid symptoms related to being monitored or “controlled” and (2) patients’ misinterpretation of interactions with the digital health tool because of paranoid delusions.

**Table 1:** Potential motivators and barriers related to patients’ use of digital health tools. For potential benefits, experts rated each item on a scale of 1 to 9, with 1=not at all likely to motivate, 5=somewhat likely to motivate, and 9=extremely likely to motivate. For potential barriers or unintended consequences, the experts rated each item from 1 to 9 with 1=significant potential, 5=some potential, and 9=minimal potential to be a barrier or unintended consequence.

Variable		Rating, mean (SD)
**Potential benefits or motivating factors^a^**		
	Improved functioning (eg, social and work functioning)	7.9 (1.3)
	Reduced symptomatology	7.8 (1.2)
	Receiving feedback or support from clinicians via the digital health system between face-to-face sessions	7.6 (1.1)
	Ability to engage with health care professionals (HCPs) periodically after discharge from face-to-face sessions	7.5 (1.1)
	Increased interaction with treatment team via digital health device in geographic areas where face-to-face access to HCPs is limited	7.4 (1.5)
	Increased confidence or self-efficacy and hope related to his or her health care	7.4 (1.3)
	Elimination or reduction of problems with transportation to treatment	7.0 (1.3)
	More personalized or tailored treatment approach can be offered by technology	7.0 (1.3)
	Receiving prompt helpful automated feedback in response to input or questions	7.0 (1.4)
**Potential barriers or unintended consequences^b^**		
	Patient does not believe that the intervention is well suited to his or her particular problem or problems	2.6 (1.8)
	Patient finds it a burden to use the digital health tool	2.6 (1.9)
	Patient does not understand how to use the digital health tool	2.8 (1.9)
	Patient has concerns about being monitored or policed	3.1 (1.9)
	Patient finds the digital health tool intrusive	3.2 (2.1)
	Patient has concerns about privacy	3.4 (1.9)
	Patient feels frustrated and discouraged with using the technology	3.5 (2.3)

^a^First-line mean ratings.

^b^Third-line mean ratings.

#### Training and Resources to Facilitate Use of Digital Health Tools by Patients

The experts rated the importance of HCPs providing different types of training and resources to help patients successfully engage with and use a digital health tool (see question 17a, Multimedia Appendix 1). A high rate of consensus was obtained on the importance of HCPs providing initial training to patients on the system, with 97% (38/39) of experts rating it first line (mean 8.4, SD 0.9). First-line ratings with a mean ≥7.4 were also given by >80% of experts to the following: responding to technical problems identified by the patients as quickly as possible, identifying trends in digital data and responding immediately to critical clinical information, providing specific and clear feedback on the data gathered via the digital health tool during treatment sessions, reinforcing patients’ continued engagement with the digital health tool within the first week of use and on an ongoing basis (independent of the clinical information reported or the outcomes), arranging for a member of the treatment team to provide ad hoc support for system use, helping patients set goals using the digital health tool, and developing reward systems for successful engagement and self-management. The experts also recommended providing follow-up and ongoing training on the system for patients (mean 7.1, SD 1.4), providing initial training on the system to families or caregivers (mean 6.9, SD 1.8), and providing specific feedback between treatment sessions about data gathered from the digital health tool (mean 6.5, SD 1.7).

The experts indicated that they believed providing many of these types of training and resources to patients would be at least somewhat difficult for the average HCP (see question 17b in Multimedia Appendix 1). Although providing initial training was rated as the most important component in helping patients successfully engage with and use a digital health tool, 56% (22/39) of experts thought that it would be somewhat difficult for HCPs to train patients (mean 5.3, SD 1.8).

### Health Care Professionals

#### Characteristics of Health Care Professionals Likely to Influence Ability to Use Digital Health Tools

The experts rated the probability that certain HCP characteristics and resources would be helpful in enabling HCPs to incorporate digital health tools in their practices (see question 2 in Multimedia Appendix 1). There was a high degree of consensus among experts (highest rating of 9 given by ≥50% of the respondents, mean rating ≥8.2) that HCPs should be enthusiastic about the tool and willing to work with patients using digital health tools, have availability of staff to support ongoing use of technology (eg, make follow-up calls and monitor progress), and have availability of necessary equipment (eg, computers and broadband internet) for delivering patient treatment on a regular basis. First-line ratings (mean and SD) were also given to the availability of staff with technology skills to train patients (mean 8.1, SD 1.1), availability of necessary equipment for patient use in the HCP’s office for training (mean 8.0, SD 1.0), HCP experience with technology (eg, computer and smartphone; mean 7.6, SD 1.0), availability of free trial versions of the tool (mean 7.5, SD 1.0), a digital health tool consistent with the HCP’s theoretical orientation (mean 7.5, SD 1.2), and a 24/7 call center to provide technical support to HCPs and patients (mean 7.4, SD 1.5).

#### Potential Benefits and Barriers or Unintended Consequences for Health Care Professionals

The experts rated potential benefits of and barriers to the use of digital health tools by HCPs who treat patients with psychiatric disorders (see questions 6 and 7 in Multimedia Appendix 1). With the assumption that the items had been demonstrated to provide benefits for HCPs, experts were asked to rate the likelihood of these items in motivating HCPs to use the tools. Reimbursement by payers for time spent training patients and family members about digital health tool and for time spent using or reviewing data from the digital health tool were the two options that received the highest rating of 9 from >60% of the experts (they were also rated first line by >90% of the experts). Improved patient functioning, reduced patient symptomatology, increased efficiency of care provision, and increased ability to deliver evidence-based treatment were among other options rated first line (all received mean rating >7.5). High second-line ratings with a mean of ≥6.8 were given to the following options: ability of multiple HCPs involved in the patient’s care to access the data (mean 7.0, SD 1.5), increased ability to tailor the treatment to a specific patient (mean 6.9, SD 1.5), 24-hour capability to assess and intervene with at-risk patients (mean 6.8, SD 2.0), and facilitation of patient disclosure about topics patients may be reluctant to share in face-to-face settings (mean 6.8, SD 1.4). No option received a third-line rating.

Drawing from the question 7 structure, using digital health tools may raise concerns for HCPs about (1) process and credibility of the intervention, (2) usability or feasibility, (3) liability and logistical issues, and (4) potential unintended consequences. Inadequate information on integrating the digital system with usual care was considered as the most significant concern regarding the process and credibility of the intervention (see Multimedia Appendix 1, question 7). Among usability issues, patients’ lack of access to and understanding of the required technology and a HCP’s perception of the digital health tool as time-consuming were considered to be potentially significant barriers in use of digital health tools by HCPs. The potential for increased liability, as well as uncertainty about receiving reimbursement, and other logistical challenges including difficulty having the digital health tool approved by insurance, time disruption involved in integrating digital data into clinical practice, and availability of too much patient information to a time-constrained HCP were deemed to have significant potential to pose barriers to use of digital health tools by HCPs. The experts did not reach consensus on whether digital health tool use could lead to the unintended consequences of patients being distracted from therapy targets or discontinuing traditional face-to-face treatment in favor of self-management.

**Table 2:** Training and resources to support health care professionals’ (HCPs’) ability to prescribe and interact with digital health tools. The experts rated each item on a scale of 1 to 9, with 1=not at all important, 5=somewhat important, and 9=extremely important.

Training or resources considered important^a^	Rating, mean (SD)
Clear rationale provided to HCPs^b^ about how using this device can improve outcomes^c^	8.3 (0.9)^c^
Provision of hands-on work with device or dashboard during training sessions	7.9 (1.2)
Inclusion of clinical examples and case materials as core elements of the training	7.9 (1.4)
Clear and concise tutorial provided in the digital device	7.7 (1.3)
Technical call center support	7.6 (1.1)
Prepared handouts to give to patients	7.4 (1.6)
In-person training sessions	7.4 (1.5)
Simple platform that can be learned with user guide and video demonstration without requiring in-person training	7.3 (1.8)
Having HCP use the digital system as a “patient” for a trial period to become familiar with its features	7.2 (1.7)
Complete protocol and user guide	7.2 (1.5)
Training provided in HCP’s office (detailing approach)	7.2 (1.7)
Availability of follow-up training sessions (if needed)	7.2 (1.4)
Continuing medical education credit for completing training	7.2 (1.6)

^a^First-line mean ratings.

^b^HCP: health care professional.

^c^Indicates options that received highest rating of 9 by ≥50% of experts.

#### Training and Resources to Facilitate Use of Digital Health Tools by Health Care Professionals

Another goal of the survey was to elicit expert opinion on the different types of training and resources that HCPs would need to successfully implement use of digital health tools in their practices (see question 16a in Multimedia Appendix 1). The experts were asked to assume that the HCP would receive computerized reports with output from the digital health tool. Table 2 shows the types of training and resources for HCPs that were considered very important (13 of the 20 listed options that were rated first line). Giving HCPs a clear rationale about how use of a digital health tool could improve outcomes was rated first line by 95% (37/39) of respondents. The types of training and resources that were considered somewhat important (high second-line ratings) included in-person training followed by Web-based video reinforcement, video-based online training modules, training webinars, frequently asked questions or user forums on a website, online chat support for questions or technical issues, and working with medical schools to provide training in digital health technology.

The experts were then asked to rate the likelihood that HCPs would participate in these types of training (see question 16b in Multimedia Appendix 1). Participation in individualized training in the HCP’s office and a concise tutorial provided in the digital health tool were rated first line. The experts thought that HCPs were somewhat likely (high second-line ratings) to use technical call center support (mean 6.4, SD 1.9), training webinars (mean 6.0, SD 1.4), follow-up training sessions when needed (mean 6.0, SD 1.7), and in-person training sessions (mean 5.9, SD 1.9). Consensus was not reached on whether HCPs would be willing to use the digital system as a “patient” for a trial period to become familiar with its features.

## Discussion

### Overview

Within the emerging field of technology in health care, a primary goal of this survey study was to provide guidance to mental health professionals (physicians and other allied HCPs such as psychologists, case managers, and social workers) on how to best integrate digital mental health tools into their practices. The presentation of the results in the preceding section was organized on the basis of target users, beginning first with considerations for patients’ use of digital health tools, followed by considerations for HCPs’ use of digital health tools. However, the following discussion is organized to show how these findings might best be applied in real-world clinical settings. The discussion will therefore begin with commentary on how to develop training and resources and how best to identify appropriate HCPs for digital health tool use, followed by a discussion of benefits that would motivate HCPs and patients, and potential barriers or unintended consequences for which one should be alert.

### Resources and Training for Health Care Professionals and Patients

Before HCPs can be asked to implement digital health tools in their practices, developers of these tools need to set up resources and training: first, to inform HCPs about *why* they might want to use such tools (ie, potential benefits of using a digital health tool for HCPs) and second, to teach interested HCPs *how* to use these devices. The experts indicated that the first and most important step in interacting with HCPs is to provide them with a clear rationale about how using a digital health tool could improve outcomes for their patients, which was rated first line by 95% (37/39) of respondents. The specific types of training for HCPs that the experts thought would be most helpful and that HCPs would be most likely to actually participate in were individualized training in their offices and a clear and concise tutorial provided in the digital health tool itself. These results suggest that strategies that are most convenient and time-efficient, particularly given the ever increasing workload demands that HCPs encounter [36], are likely to be most feasible and effective for HCPs.

Adhering to user-centered design principles to guide the development of digital health tools that are user-friendly and easy to understand is foundational to a successful digital health tool design. In the field of mental health care, provision of human support has also been recognized to be important for effective engagement with digital interventions [26]. Accordingly, Mohr et al have emphasized the “service” or support component for users in their model for designing digital health tools for mental health care [26]. Expert opinion in this survey corroborated the need for training and support to effectively engage with a digital health tool. Once the HCP has decided to begin prescribing a digital health tool and has received initial training on the device, the next and most crucial step in enabling a patient to successfully engage with and use a digital health tool is to have the HCP provide initial training to the patient (rated first line by 97% [38/39] of the experts). Other strategies that the experts indicated would be most helpful for HCPs in promoting patient engagement with a digital health tool are quick response to technical problems identified by patients, as well as critical clinical information provided by the digital health tool, provision of initial and ongoing support and reinforcement to patients by both the HCP and other members of the treatment team, and helping patients set goals using the digital health tool. However, a significant number of experts indicated that they believed carrying out these activities would be somewhat difficult for many HCPs.

### Identifying Appropriate Health Care Professionals, Motivating Them to Use Digital Health Tools, and Potential Barriers or Unintended Consequences

#### Identifying Appropriate Health Care Professionals

The experts were queried about characteristics of HCPs and resources that would increase the probability that they could successfully incorporate digital health tools in their practices. The three factors that received the highest rating from more than half of the experts and that were rated first line by ≥95% of the respondents were interest in and enthusiasm about using a digital health tool and availability of the necessary staff and equipment (eg, broadband internet if needed and mobile devices) to support use of such a tool. The experts also considered it very helpful if the HCP had experience with technology (eg, computers and smartphones).

#### Motivating Health Care Professionals

Not surprisingly, given the current insurance system in the United States, the experts rated reimbursement for time spent training patients and reviewing and using data from the digital health tool as the biggest potential motivators for HCPs to incorporate digital health tools in their practices (these options received the highest rating of 9 from >60% of the experts). The importance of addressing reimbursement and cost issues to facilitate digital health tools adoption among physicians was highlighted in a recent review by de Grood et al [24]. The experts also gave first-line ratings to many factors related to patient outcomes, including improved adherence to treatment and functioning, reduced symptomatology, more accurate data concerning adherence and symptoms on which to base clinical decision making, and a potential reduction in hospitalizations. Again, these results reinforce the importance of generating outcomes data and communicating this information to HCPs when providing initial training.

#### Potential Barriers or Unintended Consequences for Health Care Professionals

Given that digital health tools have only recently been introduced in the care of patients with SMI, we queried the experts about potential barriers to their use and about possible unintended consequences that might result from their use. The experts expressed concerns about a number of issues involving the process, usability, liability, and logistics associated with digital health tools. Data from the current survey corroborate findings of other research studies [24,37] and underscore the importance of collective industry efforts to address these issues. Not surprisingly, given the early stage of use of these technologies, the experts expressed uncertainty about a number of options that were rated as having “some” potential to be a barrier or unintended consequence but with no consensus on many of these options. It is important for HCPs in the field to remain vigilant to potential unintended consequences. Additionally, future research should examine how important these issues will actually prove to be in practice.

### Identifying Appropriate Patients, Motivating Them to Use Digital Health Tools, and Potential Barriers or Unintended Consequences

#### Identifying Appropriate Patients

As would be expected based on the treatment adherence literature [38-41], the experts endorsed positive expectations about the potential benefits of treatment, a good therapeutic alliance with the HCP, good occupational and cognitive functioning, and a readiness to change among characteristics that are very likely to promote engagement with digital health tools. The survey responses suggested that many of the characteristics that promote favorable outcomes in any psychosocial intervention are similar to those that promote favorable outcomes when interventions are delivered via a digital health tool, except that patients will also need access to and the ability to use technology.

The experts endorsed higher educational level; younger age; and interest in, access to, and familiarity with digital technology as likely to make it easier for patients to engage with and use a digital health tool, whereas sex and minority or socioeconomic status were not considered likely to affect a patient’s ability to use a digital health tool. These results correspond to those reported in studies that have investigated user characteristics related to digital health technologies. In a cross-sectional study of 100 patients with SMI, Borzekowski et al reported that sex and ethnicity did not affect the use of technology to access health information, although younger age and higher education level were associated with a greater use of the internet for this purpose [22]. More recently, Robotham et al surveyed 121 patients with psychosis and 120 with depression and found that older patients were less likely to use internet-enabled devices (eg, computers and mobile phones) [42]. In patients with psychosis, older age predicted reduced confidence in using and less access to mobile phones; in patients with depression, older age predicted reduced access to computers [42].

Although results of our survey suggest that the ability to use a digital health tool is likely independent of a specific patient diagnosis, the experts thought that specific symptomatic presentations such as poor cognitive functioning, severe symptoms, and acute substance abuse were likely to make it more difficult for patients to engage with technology. These data suggest that assessment of a patient’s clinical state and timing of a digital health tool prescription are important considerations in how clinicians employ their treatment armamentarium. A 2014 study by Gill et al, which used an automated internet-based tool to screen for depression in a sample of 45,142 individuals, reported that current depression status, previous treatment seeking for depression, and lower education level predicted lower rates of adherence with rescreening [43]. Survey results have shown that individuals who self-identify as having schizophrenia are more likely to use technologies (eg, computer, tablet, and mobile phone) when they are feeling well and not experiencing many symptoms [44]. Therefore, patients’ clinical state is an important consideration for successful use of digital health tools. Moreover, patient characteristics should be considered in the design of the digital health tool itself. For example, Rotondi and colleagues have made recommendations for mitigating the effects of cognitive dysfunction in patients with SMI [45,46].

#### Motivating Patients

HCPs will need to educate patients about potential benefits of the digital health tool in their mental health care regimen. The experts who responded to our survey endorsed a number of potential benefits that they believed would motivate patients to use a digital health tool, including improved functioning; reduced symptomatology; increased access to, interaction with, and feedback from their physician and other treatment team members; and a reduction in number of hospitalizations. The benefit associated with use of digital health tools should be empirically demonstrated by the developers of these tools so that this information can be communicated to HCPs who can then share it with their patients when introducing the digital health tool.

There was also no consensus among the experts on whether use of digital health tools might reduce health care utilization and costs. Some evidence suggests that this may be the case in other therapeutic areas. A systematic review of published literature demonstrated cost-effectiveness of mHealth interventions in areas focusing on outpatient clinic attendance, cardiovascular disease, and diabetes [47]. An ongoing randomized controlled trial aims to investigate the cost-effectiveness of an internet- and mobile-based intervention for preventing depression in individuals with chronic back pain [48]. Future research should address whether digital health tools are cost-effective in patients with SMI.

#### Potential Barriers or Unintended Consequences for Patients

Similar to the questions about potential barriers to and unintended consequences of use of digital health tools by HCPs, the experts rated a number of options as having potential to be barriers to or unintended consequences of use of digital health tools for patients (Table 1), while they also expressed uncertainty (no consensus) about many potential problems their patients might face. Future research should examine how important these issues will actually prove to be. In previous surveys of patients with schizophrenia or schizoaffective disorder, two of the most common barriers to using the internet were security concerns (46%) and lack of knowledge of technology (40%) [42].

### Study Limitations

This study had several limitations. First, the survey asked the participants to assume hypothetical use of the type of technology with which each expert had the most experience rather than asking about a specific tool or product. There is much variation in digital health tools, and the responses may have been different if the survey had specified a particular tool. Second, the expert panel represented individuals with a broad range of experience with different types of technological tools (eg, mobile phone apps, online therapy programs, and computerized cognitive remediation) and widely differing roles in treatment interventions in psychiatry. However, we achieved the goal of ensuring that the respondents were experts in the use of technology in this population. It should be noted, however, that the panel reported extensive experiences with Web or mobile or computer technologies but very little experience with sensor technologies. Third, the questions and response options specify tools that presuppose HCP involvement in digital health tool with patients. There are numerous examples of digital health tools that are consumer-facing and do not involve the HCP. Additionally, given that the experts were all developing, studying, and using technology interventions in this population, there may have been a bias to positively rate the digital health tools to advocate for their use, although experts’ own level of comfort with using technology was not assessed. Yet another limitation is that the experts’ responses may not have been well informed by first-hand experiences with digital health tools in interacting with patients in real-world settings, as indicated by the respondents spending on average approximately 15% of their professional time in clinical work. There is a potential for discrepancy between expert ratings and ratings of mental HCPs in clinical practice. Furthermore, the experts provided their perspective on patient-related factors that might differ from those of actual patients. Demographics and other characteristics of patients treated by the clinicians in this expert panel were not collected. Finally, although the survey asked about many different factors, the experts were limited to responding to predefined options.

In alignment with the emphasis on user-centered design for developing digital health tools [49], in the future, it will be important to take patient perspectives also into account. Particularly in the field of mental health care, where patients can have unique needs in engaging with technology, results of this survey can guide the choice of appropriate patients who are likely to adopt digital health tools. As patients and HCPs gain increasing experience with the use of digital health tools, future research studies and surveys in the field should seek input from actual users to facilitate effective use of digital health tools. While appreciating the potential of digital technology to transform mental health care, it will be crucial to address barriers in implementation of digital health tools and focus on efforts to make digital health tools easy to access, use, and integrate into clinical care. Various frameworks have been put forth in efforts to standardize the design process [26,46,49]; however, frameworks to harmonize the implementation and integration of digital health tools in clinical care are also needed.

### Conclusions

The use of digital health tools in the provision of mental health care is an emerging field. There is an unmet need for guidance on how to optimize the use of digital health tools to achieve desired outcomes in this patient population. In this report, we presented the recommendations of a panel of experts in this field on how to identify the most appropriate patients and HCPs to facilitate initial adoption of and sustained engagement with digital health tools and on what training and resources these target users will need. The results of this survey can guide clinicians in optimizing the use of digital health tools in psychiatry.

## Appendix

Multimedia Appendix 1 Graphical survey results.

Multimedia Appendix 2 Tabular survey results.

## Abbreviations

HCP: health care professional
MDD: major depressive disorder
mHealth: mobile health
SMI: serious mental illness

## References

[1] Ngui EM, Khasakhala L, Ndetei D, Roberts LW (2010). Mental disorders, health inequalities and ethics: a global perspective. Int Rev Psychiatry.

[2] US Food and Drug Administration.

[3] Klein S, Hostetter M, McCarthy D (2014). The Commonwealth Fund.

[4] Huxley CJ, Atherton H, Watkins JA, Griffiths F (2015). Digital communication between clinician and patient and the impact on marginalised groups: a realist review in general practice. Br J Gen Pract.

[5] McAuley A (2014). Digital health interventions: widening access or widening inequalities?. Public Health.

[6] Block G, Azar KM, Romanelli RJ, Block TJ, Hopkins D, Carpenter HA, Dolginsky MS, Hudes ML, Palaniappan LP, Block CH (2015). Diabetes prevention and weight loss with a fully automated behavioral intervention by email, web, and mobile phone: a randomized controlled trial among persons with prediabetes. J Med Internet Res.

[7] Martin SS, Feldman DI, Blumenthal RS, Jones SR, Post WS, McKibben RA, Michos ED, Ndumele CE, Ratchford EV, Coresh J, Blaha MJ (2015). mActive: a randomized clinical trial of an automated mHealth intervention for physical activity promotion. J Am Heart Assoc.

[8] Quinn CC, Shardell MD, Terrin ML, Barr EA, Ballew SH, Gruber-Baldini AL (2011). Cluster-randomized trial of a mobile phone personalized behavioral intervention for blood glucose control. Diabetes Care.

[9] Andersson G (2009). Using the internet to provide cognitive behaviour therapy. Behav Res Ther.

[10] Carlbring P, Bohman S, Brunt S, Buhrman M, Westling BE, Ekselius L, Andersson G (2006). Remote treatment of panic disorder: a randomized trial of internet-based cognitive behavior therapy supplemented with telephone calls. Am J Psychiatry.

[11] Mohr DC, Burns MN, Schueller SM, Clarke G, Klinkman M (2013). Behavioral intervention technologies: evidence review and recommendations for future research in mental health. Gen Hosp Psychiatry.

[12] Ben-Zeev D, Scherer EA, Gottlieb JD, Rotondi AJ, Brunette MF, Achtyes ED, Mueser KT, Gingerich S, Brenner CJ, Begale M, Mohr DC, Schooler N, Marcy P, Robinson DG, Kane JM (2016). mHealth for schizophrenia: patient engagement with a mobile phone intervention following hospital discharge. JMIR Ment Health.

[13] Faurholt-Jepsen M, Frost M, Vinberg M, Christensen EM, Bardram JE, Kessing LV (2014). Smartphone data as objective measures of bipolar disorder symptoms. Psychiatry Res.

[14] Granholm E, Ben-Zeev D, Link PC, Bradshaw KR, Holden JL (2012). Mobile Assessment and Treatment for Schizophrenia (MATS): a pilot trial of an interactive text-messaging intervention for medication adherence, socialization, and auditory hallucinations. Schizophr Bull.

[15] Ben-Zeev D, Brenner CJ, Begale M, Duffecy J, Mohr DC, Mueser KT (2014). Feasibility, acceptability, and preliminary efficacy of a smartphone intervention for schizophrenia. Schizophr Bull.

[16] Karyotaki E, Riper H, Twisk J, Hoogendoorn A, Kleiboer A, Mira A, Mackinnon A, Meyer B, Botella C, Littlewood E, Andersson G, Christensen H, Klein JP, Schröder J, Bretón-López J, Scheider J, Griffiths K, Farrer L, Huibers MJ, Phillips R, Gilbody S, Moritz S, Berger T, Pop V, Spek V, Cuijpers P (2017). Efficacy of self-guided internet-based cognitive behavioral therapy in the treatment of depressive symptoms: a meta-analysis of individual participant data. JAMA Psychiatry.

[17] Motter JN, Pimontel MA, Rindskopf D, Devanand DP, Doraiswamy PM, Sneed JR (2016). Computerized cognitive training and functional recovery in major depressive disorder: a meta-analysis. J Affect Disord.

[18] Wykes T, Huddy V, Cellard C, McGurk SR, Czobor P (2011). A meta-analysis of cognitive remediation for schizophrenia: methodology and effect sizes. Am J Psychiatry.

[19] Rotondi AJ, Anderson CM, Haas GL, Eack SM, Spring MB, Ganguli R, Newhill C, Rosenstock J (2010). Web-based psychoeducational intervention for persons with schizophrenia and their supporters: one-year outcomes. Psychiatr Serv.

[20] Wenze SJ, Armey MF, Miller IW (2014). Feasibility and acceptability of a mobile intervention to improve treatment adherence in bipolar disorder: a pilot study. Behav Modif.

[21] Ben-Zeev D, Davis KE, Kaiser S, Krzsos I, Drake RE (2013). Mobile technologies among people with serious mental illness: opportunities for future services. Adm Policy Ment Health.

[22] Borzekowski DL, Leith J, Medoff DR, Potts W, Dixon LB, Balis T, Hackman AL, Himelhoch S (2009). Use of the internet and other media for health information among clinic outpatients with serious mental illness. Psychiatr Serv.

[23] Nicholas J, Huckvale K, Larsen ME, Basu A, Batterham PJ, Shaw F, Sendi S (2017). Issues for eHealth in psychiatry: results of an expert survey. J Med Internet Res.

[24] de Grood C, Raissi A, Kwon Y, Santana MJ (2016). Adoption of e-health technology by physicians: a scoping review. J Multidiscip Healthc.

[25] (2015). IMS Institute for Healthcare Informatics.

[26] Mohr DC, Lyon AR, Lattie EG, Reddy M, Schueller SM (2017). Accelerating digital mental health research from early design and creation to successful implementation and sustainment. J Med Internet Res.

[27] Muuraiskangas S, Harjumaa M, Kaipainen K, Ermes M (2016). Process and effects evaluation of a digital mental health intervention targeted at improving occupational well-being: lessons from an intervention study with failed adoption. JMIR Ment Health.

[28] Torous J, Staples P, Slaters L, Adams J, Sandoval L, Onnela JP, Keshavan M (2017). Characterizing smartphone engagement for schizophrenia: results of a naturalist mobile health study. Clin Schizophr Relat Psychoses.

[29] Kvedar JC, Fogel AL (2017). https://catalyst.nejm.org/real-world-results-digital-health-products/.

[30] Yardley L, Spring BJ, Riper H, Morrison LG, Crane DH, Curtis K, Merchant GC, Naughton F, Blandford A (2016). Understanding and promoting effective engagement with digital behavior change interventions. Am J Prev Med.

[31] Kahn DA, Docherty JP, Carpenter D, Frances A (1997). Consensus methods in practice guideline development: a review and description of a new method. Psychopharmacol Bull.

[32] Altshuler LL, Cohen LS, Moline ML, Kahn DA, Carpenter D, Docherty JP, Expert Consensus Panel for Depression in Women (2001). The Expert Consensus Guideline Series. Treatment of depression in women. Postgrad Med.

[33] (1996). Treatment of bipolar disorder. The Expert Consensus Panel for Bipolar Disorder. J Clin Psychiatry.

[34] Velligan DI, Weiden PJ, Sajatovic M, Scott J, Carpenter D, Ross R, Docherty JP, Expert Consensus Panel on Adherence Problems in Serious and Persistent Mental Illness (2009). The expert consensus guideline series: adherence problems in patients with serious and persistent mental illness. J Clin Psychiatry.

[35] Brook RH, Chassin MR, Fink A, Solomon DH, Kosecoff J, Park RE (1986). A method for the detailed assessment of the appropriateness of medical technologies. Int J Technol Assess Health Care.

[36] Hobbs FDR, Bankhead C, Mukhtar T, Stevens S, Perera-Salazar R, Holt T, Salisbury C, National Institute for Health Research School for Primary Care Research (2016). Clinical workload in UK primary care: a retrospective analysis of 100 million consultations in England, 2007-14. Lancet.

[37] (2016). American Medical Association.

[38] Karow A, Czekalla J, Dittmann RW, Schacht A, Wagner T, Lambert M, Schimmelmann BG, Naber D (2007). Association of subjective well-being, symptoms, and side effects with compliance after 12 months of treatment in schizophrenia. J Clin Psychiatry.

[39] Lacro JP, Dunn LB, Dolder CR, Leckband SG, Jeste DV (2002). Prevalence of and risk factors for medication nonadherence in patients with schizophrenia: a comprehensive review of recent literature. J Clin Psychiatry.

[40] McCabe R, Bullenkamp J, Hansson L, Lauber C, Martinez-Leal R, Rössler W, Salize HJ, Svensson B, Torres-Gonzalez F, van den Brink R, Wiersma D, Priebe S (2012). The therapeutic relationship and adherence to antipsychotic medication in schizophrenia. PLoS One.

[41] Novick D, Haro JM, Suarez D, Perez V, Dittmann RW, Haddad PM (2010). Predictors and clinical consequences of non-adherence with antipsychotic medication in the outpatient treatment of schizophrenia. Psychiatry Res.

[42] Robotham D, Satkunanathan S, Doughty L, Wykes T (2016). Do we still have a digital divide in mental health? A five-year survey follow-up. J Med Internet Res.

[43] Gill S, Contreras O, Muñoz RF, Leykin Y (2014). Participant retention in an automated online monthly depression rescreening program: patterns and predictors. Internet Interv.

[44] Gay K, Torous J, Joseph A, Pandya A, Duckworth K (2016). Digital technology use among individuals with schizophrenia: results of an online survey. JMIR Ment Health.

[45] Rotondi AJ, Sinkule J, Haas GL, Spring MB, Litschge CM, Newhill CE, Ganguli R, Anderson CM (2007). Designing websites for persons with cognitive deficits: design and usability of a psychoeducational intervention for persons with severe mental illness. Psychol Serv.

[46] Rotondi AJ, Eack SM, Hanusa BH, Spring MB, Haas GL (2015). Critical design elements of e-health applications for users with severe mental illness: singular focus, simple architecture, prominent contents, explicit navigation, and inclusive hyperlinks. Schizophr Bull.

[47] Iribarren SJ, Cato K, Falzon L, Stone PW (2017). What is the economic evidence for mHealth? A systematic review of economic evaluations of mHealth solutions. PLoS One.

[48] Sander L, Paganini S, Lin J, Schlicker S, Ebert DD, Buntrock C, Baumeister H (2017). Effectiveness and cost-effectiveness of a guided internet- and mobile-based intervention for the indicated prevention of major depression in patients with chronic back pain-study protocol of the PROD-BP multicenter pragmatic RCT. BMC Psychiatry.

[49] Baek E, Cagiltay K, Boling E, Frick T, Spector JM, Merrill MD, van Merrienboer J, Driscoll MP (2007). User-centered design and development. Handbook of Research on Educational Communications and Technology. Third edition.

