# Supplementary material for: Expert Consensus Survey on Digital Health Tools for Patients With Serious Mental Illness: Optimizing for User Characteristics and User Support
Source: JMIR Ment Health. 2018 Jun 12;5(2):e46. doi: 10.2196/mental.9777 (PMC6019847; doi:10.2196/mental.9777)
Supplement: Multimedia Appendix 1 [file mental_v5i2e46_app1.pdf]

Expert Consensus Survey on Digital Health Tools 2015

Question 1. **Characteristics of PATIENTS that affect ability to engage with and use a digital health tool**. How much do you believe the following patient characteristics affect the chances that a patient can successfully engage with and use a digital health tool? In answering this and subsequent questions, please consider the type of digital health tool(s) with which you have the most experience. (Please rate each item on a scale from 1 to 9 with: 1 = likely to make it very difficult for person to engage with and use, 5 = not likely to influence ability to engage with or use, and 9 = extremely likely to promote engagement and ability to use.)

1a. Patient Characteristics

	95% Confidence Intervals (Third Line / Second Line / First Line)	*N*	Avg (SD)	*Tr of Chc*	1st Line	2nd Line	3rd Line
Interest in using state-of-the-art technology	*	39	8.0 (1.5)	*54*	90	8	3
Resources that facilitate access to treatment (availability of Wi-Fi or data plan, applicable hardware, insurance coverage)		40	7.8 (1.0)	*25*	93	8	0
Positive expectations on the part of the patient about using a digital health tool		40	7.8 (1.0)	*28*	90	10	0
Owns and uses a smart phone/computer/tablet		40	7.8 (0.9)	*23*	90	10	0
Positive social support (i.e., patient has an involved significant other or caregiver)		40	7.4 (0.9)	*10*	90	10	0
< 18 years old		40	7.3 (1.8)	*33*	78	18	5
18-44 years old		40	7.3 (1.2)	*18*	73	28	0
Bachelor's degree or more education		40	7.0 (1.0)	*5*	70	30	0
High school or more education		40	6.1 (1.1)	*0*	43	55	3
Female gender		40	5.6 (1.1)	*3*	23	78	0
45-64 years old		40	5.6 (1.2)	*0*	28	70	3
Currently married		40	5.5 (0.9)	*0*	15	85	0
Male gender		40	5.5 (1.2)	*3*	23	73	5
Minority status		40	4.8 (1.0)	*0*	5	85	10
Low socioeconomic status		40	4.0 (1.2)	*0*	5	60	35
Did not complete high school		40	3.8 (1.0)	*0*	0	63	38
65+ years old		40	3.7 (1.5)	*0*	5	43	53
Low health literacy		40	3.4 (1.1)	*0*	0	43	58
Severe psychosocial stressors (e.g., poverty, general medical problems, abusive relationship, legal problems)		40	2.9 (1.2)	*0*	0	28	73
Low IQ		40	2.9 (0.8)	*0*	0	18	83
None or minimal knowledge/ability/comfort using technology (e.g., computers, tablets, mobile phones)		39	2.7 (0.9)	*0*	0	15	85
Low literacy (i.e., reading ability)/numeracy		40	2.4 (0.8)	*0*	0	5	95
Low motivation		40	2.2 (0.9)	*0*	0	5	95
Serious level of chaos and disorganization in the person's life/environment		40	2.1 (0.9)	*0*	0	8	93
	1 2 3 4 5 6 7 8 9			*%*	%	%	%

1b. Disorders, Signs, and Symptoms

	95% Confidence Intervals: Third Line / Second Line / First Line	*N*	Avg (SD)	*Tr of Chc*	1st Line	2nd Line	3rd Line
Good occupational functioning		39	6.9 (1.1)	*3*	74	26	0
Diagnosis of bipolar disorder		40	5.2 (1.1)	*3*	10	85	5
Diagnosis of major depressive disorder		40	5.0 (1.3)	*3*	13	78	10
Non-suicidal self-injurious behaviors		40	5.0 (0.6)	*0*	3	98	0
Suicidal ideation		40	4.7 (1.2)	*0*	3	80	18
Multiple previous episodes		40	4.6 (1.0)	*0*	0	88	13
Diagnosis of schizophrenia		40	4.5 (1.2)	*0*	5	75	20
Longer duration of illness		40	4.5 (1.3)	*0*	5	73	23
Sensitivity to overstimulation		40	4.2 (1.3)	*0*	5	65	30
Presence of Axis II comorbidity		40	4.1 (1.1)	*0*	0	75	25
Multiple comorbid Axis I disorders		40	3.9 (1.2)	*0*	0	60	40
Greater severity of delusions/paranoia		40	3.6 (1.3)	*0*	0	53	48
Aggression/agitation		40	3.5 (1.3)	*0*	0	55	45
Acute substance use		39	3.3 (1.3)	*0*	0	49	51
Low energy/fatigue		40	3.3 (0.9)	*0*	0	45	55
Low frustration tolerance		40	3.0 (0.9)	*0*	0	28	73
Greater severity of negative symptoms		39	2.9 (1.4)	*0*	0	31	69
Greater severity of disorganization symptoms		40	2.9 (1.3)	*0*	3	25	73
Greater severity of neurocognitive impairment		40	2.8 (1.2)	*0*	0	28	73
	1 2 3 4 5 6 7 8 9			*%*	%	%	%

1c. Appraisals and Patient Experience

95% CONFIDENCE INTERVALS: Third Line, Second Line, First Line

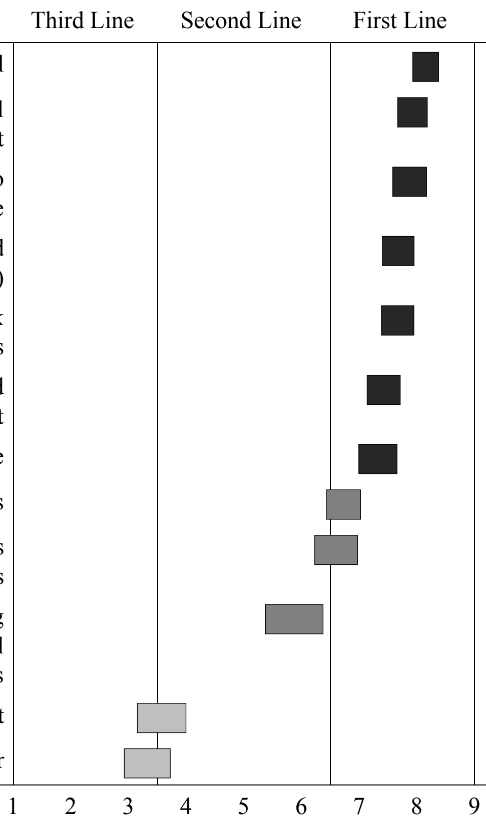

	N	Avg (SD)	*Tr of Chc*	1st Line	2nd Line	3rd Line
Perception of digital treatment as beneficial	39	8.2 (0.7)	*33*	100	0	0
Agreement with tasks and goals of digital treatment	40	7.9 (0.8)	*28*	98	3	0
Self-efficacy beliefs about being able to use/operate the device	40	7.9 (0.9)	*28*	90	10	0
Positive bond with healthcare professional (good therapeutic alliance)	40	7.7 (0.9)	*18*	90	10	0
Willingness to complete tasks/homework between treatment sessions	39	7.7 (0.9)	*18*	90	10	0
History of good adherence to medication and other treatment	40	7.4 (0.9)	*15*	85	15	0
Readiness to change	40	7.3 (1.1)	*13*	78	23	0
Psychological mindedness	40	6.7 (1.0)	*0*	63	38	0
Engagement with mutual help organizations and/or peer support groups	40	6.6 (1.2)	*5*	50	50	0
Stigma/discomfort/social anxiety about seeking face-to-face care for mental or behavioral problems	40	5.9 (1.6)	*5*	35	55	10
Negative past experience with treatment	40	3.6 (1.4)	*0*	0	53	48
Limited insight into disorder	40	3.3 (1.3)	*0*	0	43	58
			%	%	%	%

Question 2. Characteristics and Resources of HEALTH CARE PROFESSIONALS (HCPs). How helpful do you believe the following characteristics and resources are in enabling HCPs to successfully incorporate digital health tools in their practices? (Please rate each item on a scale from 1 to 9 with 1 = not at all helpful 5 = somewhat helpful 9 = extremely helpful)

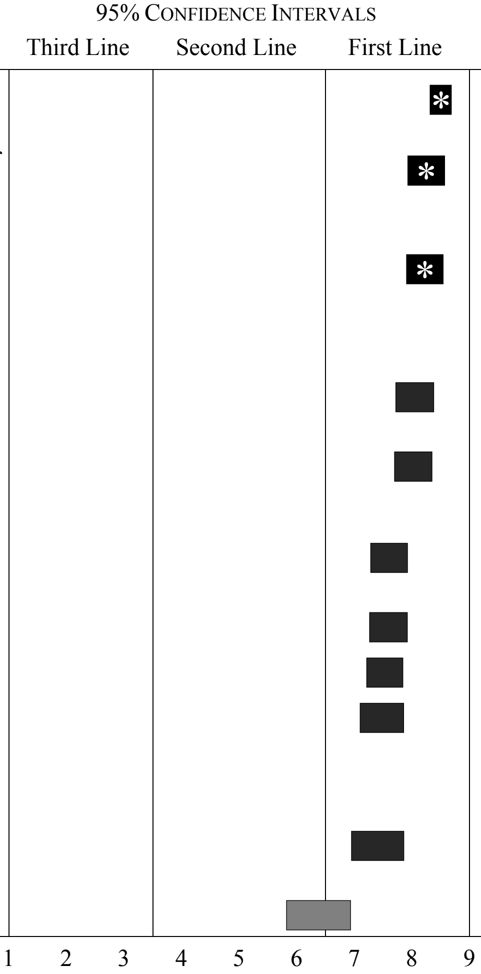

	N	Avg (SD)	*Tr of Chc*	1st Line	2nd Line	3rd Line
HCP enthusiastic about/willing to work with patients using digital health tools	40	8.5 (0.6)	*55*	100	0	0
Availability of staff to support the ongoing use of the prescribed technology (e.g. make follow-up phone calls, monitor progress)	40	8.3 (1.0)	*50*	95	5	0
Availability of necessary equipment (e.g. computers, broadband internet if needed, mobile devices) for delivering patient treatment on a regular basis	40	8.2 (1.0)	*50*	95	5	0
Availability of staff with technology skills to teach patients how to use technology initially	40	8.1 (1.1)	*40*	95	5	0
Availability of necessary equipment (e.g. computers, broadband internet if needed, mobile devices) for patient use in the office for training	40	8.0 (1.0)	*40*	90	10	0
HCP is experienced in using computers in practice	40	7.6 (1.0)	*25*	85	15	0
HCP has familiarity with smart phone technology	39	7.6 (1.0)	*26*	85	15	0
Availability of free trial version of tools	40	7.5 (1.0)	*13*	88	13	0
Availability of a digital health tool that is consistent with the HCP's preferred theoretical orientation (e.g., a digital CBT tool for a therapist trained in CBT)	40	7.5 (1.2)	*18*	80	20	0
Availability of 24/7 call center to provide technical support for HCP and patients	40	7.4 (1.5)	*23*	80	18	3
HCP is ≤ 40 years old	40	6.4 (1.8)	*10*	58	35	8
			%	%	%	%

Question 4. Potential benefits of using a digital health tool for PATIENTS. Assume the items below have been demonstrated to be benefits of using a digital health tool for patients with psychiatric disorders. For each, please rate how likely you believe it would be to motivate a patient to use the digital health tool. (Please rate each item on a scale from 1 to 9 with 1 = not at all likely to motivate patient 5 = somewhat likely to motivate patient 9 = extremely likely to motivate patient)

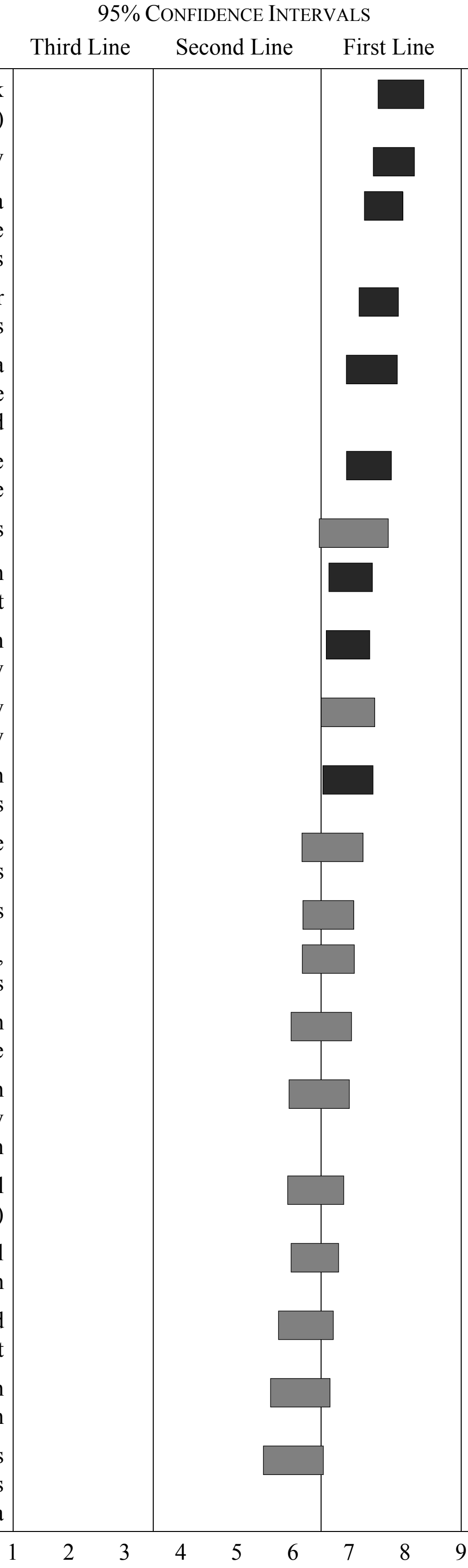

	N	Avg (SD)	*Tr of Chc* %	1st Line %	2nd Line %	3rd Line %
Improved functioning (e.g., social and work functioning)	40	7.9 (1.3)	*38*	93	5	3
Reduced symptomatology	40	7.8 (1.2)	*30*	83	18	0
Receiving feedback/support from clinicians via the digital health system between face-to-face sessions	39	7.6 (1.1)	*21*	85	15	0
Ability to engage with HCP periodically after discharge from face-to-face sessions	40	7.5 (1.1)	*18*	78	23	0
Increased interaction with treatment team via digital health device in geographic areas where face-to-face access to HCPs is limited	40	7.4 (1.5)	*25*	78	20	3
Increased confidence/self-efficacy and hope related to his or her health care	40	7.4 (1.3)	*23*	75	23	3
Reduction in number of hospitalizations	38	7.1 (1.9)	*26*	76	16	8
Elimination or reduction of problems with transportation to treatment	40	7.0 (1.3)	*10*	68	33	0
More personalized/tailored treatment approach can be offered by technology	40	7.0 (1.3)	*3*	70	30	0
Increased social engagement enabled by technology	40	7.0 (1.5)	*18*	70	28	3
Receiving prompt helpful automated feedback in response to input/questions	40	7.0 (1.4)	*10*	70	28	3
Increased willingness to engage in care for those who are reluctant to meet face-to-face with HCPs	40	6.7 (1.8)	*13*	63	33	5
Access to state-of the art treatment tools	40	6.6 (1.5)	*10*	50	48	3
Better self-understanding of thinking, feelings, and behaviors	40	6.6 (1.5)	*5*	60	38	3
Decreased stigma related to seeking mental health care	40	6.5 (1.7)	*10*	60	35	5
Improved communication/understanding between patients and family members because of family integration in digital system	39	6.5 (1.7)	*8*	54	38	8
Self-management and ability to record personal information (e.g., about adherence, symptoms)	40	6.4 (1.6)	*8*	45	48	8
Around the clock access to a digital health tool that is part of the patient's treatment plan	39	6.4 (1.3)	*3*	46	51	3
Digital links to wide range of resources and support	40	6.2 (1.6)	*0*	53	43	5
Increased understanding and investment in treatment plan	40	6.1 (1.7)	*8*	45	48	8
Ability of multiple HCPs involved in patient's care (e.g., case manager, psychologist) to access the data	40	6.0 (1.7)	*8*	43	50	8

Question 4. *Continued*

95% CONFIDENCE INTERVALS (Third Line 1–3, Second Line 4–6, First Line 7–9)

	N	Avg (SD)	*Tr of Chc* %	1st Line %	2nd Line %	3rd Line %
Improved patient adherence to medication and treatment plan	39	5.7 (1.7)	*0*	38	46	15
Potential to reduce utilization of health care services and therefore cost of treatment	40	5.6 (2.1)	*5*	40	40	20
Media representation more engaging than face-to-face interactions	40	5.3 (1.8)	*0*	28	53	20

Question 5. Potential barriers/unintended consequences when using digital health tool (e.g., mobile health application, computerized treatment tool, website). Considering average patient with serious mental illness (schizophrenia, bipolar, or major depressive disorder), how likely do you believe each of the items below is to be a potential barrier and/or to be or lead to an unintended consequence for PATIENTS using a digital health tool? (Please rate each item on a scale from 1 to 9 with 1 = significant potential to be a barrier and/or an unintended consequence 5 = some potential to be a barrier and/or an unintended consequence 9 = minimal potential to be a barrier and/or an unintended consequence)

95% CONFIDENCE INTERVALS (Third Line 1–3, Second Line 4–6, First Line 7–9)

	N	Avg (SD)	*Tr of Chc* %	1st Line %	2nd Line %	3rd Line %
Increase in family conflict due to information provided by the digital tool	39	5.5 (2.4)	*8*	46	28	26
Depersonalization of patient care and potential damage to the therapeutic relationship	40	5.0 (2.5)	*8*	35	30	35
Patient's use of digital health tool in isolation leads to decreased face-to-face interaction with clinician and/or treatment team	40	4.8 (2.3)	*0*	30	38	33
Patient sets unrealistic goal and becomes discouraged	39	4.4 (2.5)	*3*	31	21	49
Patient disappointed by not receiving prompt response from HCP	40	4.3 (1.9)	*0*	20	35	45
Patient feels frustrated and discouraged with using the technology	40	3.5 (2.3)	*3*	15	23	63
Patient has concerns about privacy	40	3.4 (1.9)	*0*	8	33	60
Patient finds the digital health tool intrusive	40	3.2 (2.1)	*0*	15	13	73
Patient has concerns about being monitored or policed	40	3.1 (1.9)	*3*	8	25	68
Patient does not understand how to use the digital health tool	40	2.8 (1.9)	*3*	5	23	73
Patient finds it a burden to use the digital health tool	40	2.6 (1.9)	*3*	8	10	83
Patient does not believe that the intervention is well suited to his/her particular problem(s).	40	2.6 (1.8)	*3*	8	10	83
For following 2 items, consider only patients with psychosis:	0	0.0 (0.0)	*0*	0	0	0
Possible exacerbation of paranoid symptoms related to being monitored or "controlled"	40	4.8 (2.7)	*3*	38	28	35
Patient misinterprets interactions with the digital health tool because of paranoid delusions	40	4.7 (2.7)	*3*	35	25	40

Question 6. Potential benefits of using a digital health tool for HEALTH CARE PROFESSIONALS. Assume the items below have been demonstrated to be benefits of using a digital health tool for HCPs who treat patients with psychiatric disorders. For each, please rate how likely you believe it would be to motivate an HCP to use the digital health tool. (Please rate each item on a scale from 1 to 9 with 1 = not at all likely to motivate HCP 5 = somewhat likely to motivate HCP 9 = extremely likely to motivate HCP)

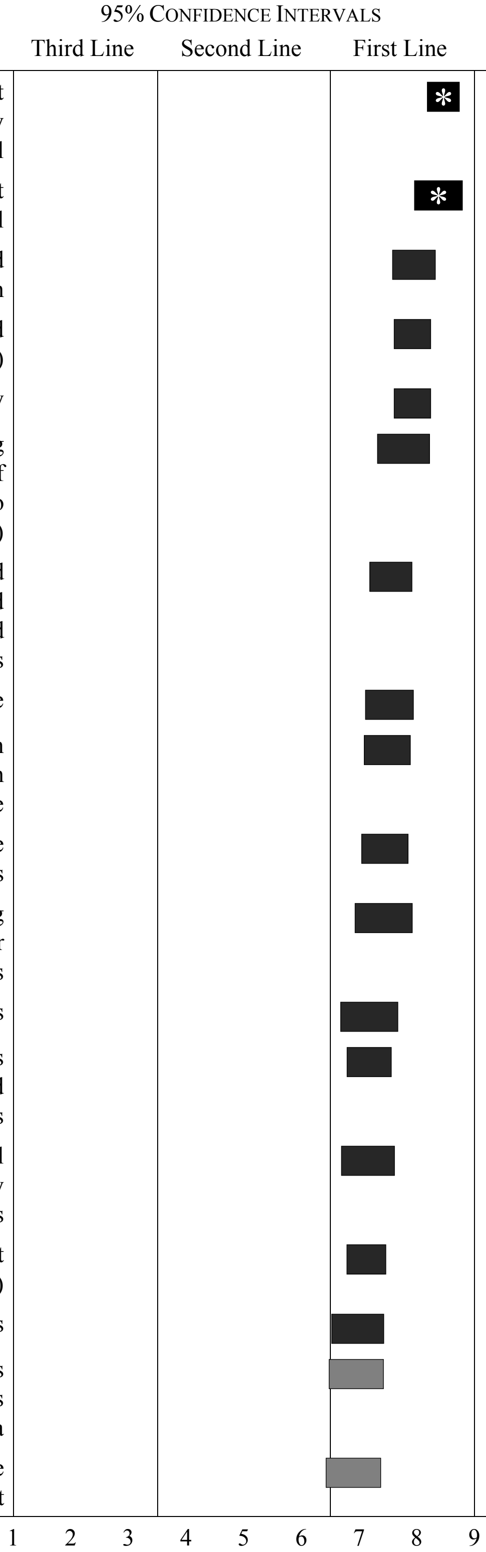

	N	Avg (SD)	*Tr of Chc*	1st Line	2nd Line	3rd Line
Reimbursement by payers for time spent providing training to patients and family members about the digital health tool	39	8.5 (0.9)	*62*	95	5	0
Reimbursement by payers for time spent using/reviewing data from the digital health tool	40	8.4 (1.3)	*65*	93	5	3
Improved patient adherence to medication and treatment plan	40	8.0 (1.2)	*38*	93	5	3
Improved patient functioning (e.g., social and work functioning)	39	7.9 (1.0)	*36*	90	10	0
Reduced patient symptomatology	39	7.9 (1.0)	*33*	92	8	0
Increased efficiency of care provision by freeing up HCP's time without compromising quality of care (i.e., technology as a treatment extender to reduce HCP burden)	39	7.8 (1.4)	*38*	87	10	3
Increased ability to deliver evidence-based treatments that would not otherwise be delivered because of limited staff resources, limited trained staff, or cost barriers	40	7.6 (1.2)	*20*	80	20	0
More accurate data about medication adherence	40	7.5 (1.3)	*20*	85	13	3
Digital health tool provides input to common electronic health record (EHR) systems already in use	39	7.5 (1.3)	*26*	85	15	0
Published evidence concerning efficacy of the digital health tool in improving patient outcomes	38	7.4 (1.3)	*21*	76	24	0
Replacing routine assessment and monitoring functions, thus increasing availability of HCP for more complex cases	40	7.4 (1.6)	*28*	85	10	5
Reduction in number of hospitalizations	40	7.2 (1.6)	*25*	70	28	3
Potential to achieve improved patient outcomes by being able to make more evidence-based treatment decisions	40	7.2 (1.2)	*18*	75	25	0
Improved accuracy of information for clinical decision-making based on objective momentary data about patient symptoms	39	7.2 (1.5)	*21*	69	31	0
Access to more patient information (e.g., about adherence, symptoms)	40	7.1 (1.1)	*13*	70	30	0
Ability to provide state-of-the art treatment tools	40	7.0 (1.5)	*13*	70	28	3
Ability of multiple HCPs involved in patient's care (e.g., case manager, psychologist) to access the data	40	7.0 (1.5)	*15*	63	35	3
Increased ability to personalize/tailor the treatment approach to the specific patient	40	6.9 (1.5)	*15*	58	40	3
			%	%	%	%

Question 6. *Question*

95% CONFIDENCE INTERVALS

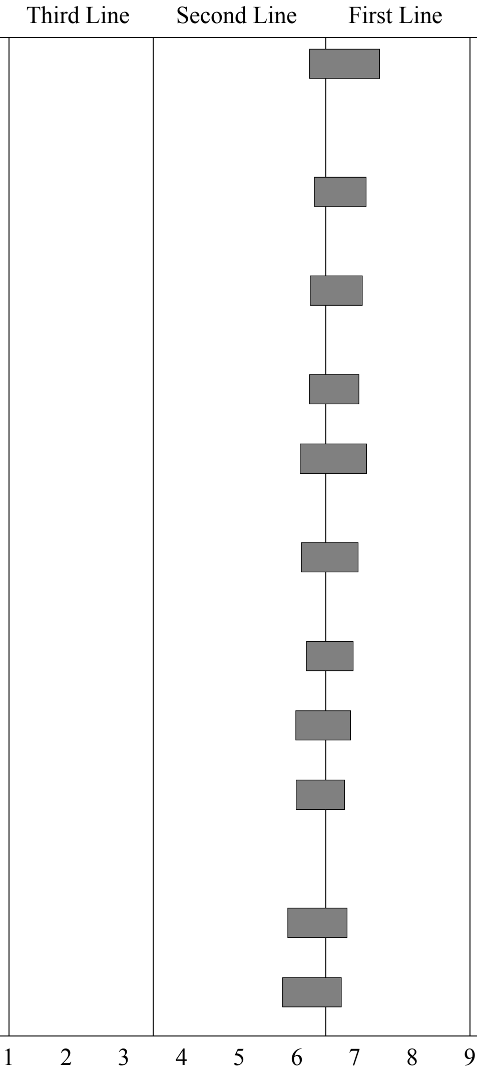

	N	Avg (SD)	*Tr of Chc*	1st Line	2nd Line	3rd Line
Around the clock capability to assess and intervene with at-risk patients (e.g., with suicidal risk or increased agitation/aggression) in a way that may increase safety	40	6.8 (2.0)	*25*	63	30	8
Facilitation of patient disclosure about topics that patients may be reluctant to share in traditional face-to-face settings	40	6.8 (1.4)	*5*	65	30	5
Improved accuracy of information for clinical decision-making based on objective sensor data (e.g., external biometric sensors, GPS, Bluetooth)	40	6.7 (1.5)	*10*	60	40	0
Increased patient social engagement enabled by technology	39	6.6 (1.4)	*5*	56	41	3
Around the clock capability to to identify potential problems/early warning signs of acute episodes in order to intervene more quickly	40	6.6 (1.9)	*15*	58	35	8
Potential to reduce utilization of health care services (e.g., emergency, outpatient) and therefore cost of treatment	39	6.6 (1.6)	*8*	59	33	8
Better understanding of patient's thinking, feelings, and behaviors	39	6.6 (1.3)	*0*	67	28	5
Increased confidence/self-efficacy related to the patient's health care	40	6.5 (1.5)	*5*	55	40	5
Improved communication/understanding between patients and family members because of family integration in digital system (assuming this is a goal of treatment)	40	6.4 (1.4)	*5*	53	45	3
Opportunity to provide feedback/support to patients between face-to-face sessions	40	6.4 (1.7)	*3*	53	40	8
Ability to engage periodically with patients after discharge from face-to-face sessions	39	6.3 (1.6)	*8*	44	49	8
			%	%	%	%

Question 7. Potential barriers and/or unintended consequences for HEALTH CARE PROFESSIONALS. Listed below are some potential barriers to and unintended consequences of HCPs' use of a digital health tool with patients with psychiatric disorders. How likely do you believe each of the items is to be a potential barrier and/or to be or lead to an unintended consequence for HCPs using a digital health tool with their patients? (Please rate each item on a scale from 1 to 9 with 1 = significant potential to be a barrier and/or an unintended consequence for an HCP 5 = some potential to be a barrier and/or an unintended consequence for an HCP 9 = minimal potential to be a barrier and/or an unintended consequence for an HCP)

7a. Concerns about process and credibility of intervention

	95% Confidence Intervals: Third Line (1–3) / Second Line (4–6) / First Line (7–9)	*N*	Avg (SD)	*Tr of Chc* %	1st Line %	2nd Line %	3rd Line %
Loss of expert role due to inadequate technological knowledge		40	4.7 (2.1)	*3*	23	43	35
Concern about validation of measures		40	4.5 (2.0)	*3*	18	50	33
Concern about preserving the validity of evidence-based psychosocial interventions when delivered digitally		40	4.4 (2.4)	*3*	28	30	43
Uncertainty about how to discuss and explain the digital health tool to patients		40	4.4 (2.6)	*3*	28	23	50
Potential for blurring of patient-professional boundaries (e.g., digital tools allow contact outside scheduled face-to-face appointments)		40	4.4 (2.2)	*0*	23	30	48
Lack of centralized regulation/approval/rating body for novel technologies leads to unclear expected benefits		40	4.3 (2.0)	*3*	18	40	43
Inadequate information about how the processes delivered by digital systems can be integrated with in-person, usual care		40	3.0 (1.8)	*0*	8	18	75
	1 2 3 4 5 6 7 8 9			*%*	%	%	%

7b. Concerns about usability/feasibility

	95% Confidence Intervals: Third Line (1–3) / Second Line (4–6) / First Line (7–9)	*N*	Avg (SD)	*Tr of Chc* %	1st Line %	2nd Line %	3rd Line %
Concern that the tool has not gone through sufficient usability testing with psychiatric population		40	3.5 (1.6)	*0*	5	33	63
HCP has doubts about the patient's ability to handle the required technology		40	2.3 (1.4)	*0*	3	10	88
HCP considers the device a hassle/too time consuming to appropriately bring in to care		40	2.1 (1.3)	*0*	0	13	88
Patient does not have access to the required technology		40	1.9 (1.3)	*0*	3	5	93
	1 2 3 4 5 6 7 8 9			*%*	%	%	%

7c. Liability and logistical issues

	95% Confidence Intervals: Third Line	Second Line	First Line	*N*	Avg (SD)	*Tr of Chc*	1st Line	2nd Line	3rd Line
Concern about patient confidentiality				40	3.5 (1.8)	*0*	10	25	65
Uncertainty about the frequency with which the digital data should be reviewed				40	3.5 (1.6)	*0*	3	45	53
Uncertainty about how to use the information clinically				40	3.2 (1.5)	*0*	3	40	58
Availability of too much information (e.g., HCP overwhelmed with additional patient information that needs consideration despite time-pressured schedule)				40	2.6 (1.6)	*0*	3	15	83
Time/disruption involved in integrating digital data into clinical practice				40	2.5 (1.2)	*0*	0	20	80
Difficulty having the digital health tool approved by insurance				40	2.4 (1.5)	*0*	3	15	83
Uncertainty about receiving reimbursement for time spent training or using the device and its data				40	2.3 (1.4)	*0*	0	18	83
Concern about potential for increased liability exposure due to not having effected or documented a clinical response to available information				39	2.2 (1.3)	*0*	0	13	87
	1 2 3	4 5 6	7 8 9			*%*	%	%	%

7d. Unintended consequences

	95% Confidence Intervals: Third Line	Second Line	First Line	*N*	Avg (SD)	*Tr of Chc*	1st Line	2nd Line	3rd Line
Patient discontinues needed traditional face-to-face treatment in favor of self-management				40	5.0 (2.3)	*3*	33	38	30
Distraction from therapy targets and tasks identified in session				40	4.7 (1.9)	*0*	23	50	28
	1 2 3	4 5 6	7 8 9			*%*	%	%	%

Question 16. **Training for Health Care Professionals (HCPs)**. In the next two questions, we ask you to rate a number of different types of training and training resources for HCPs. In 16a, we ask you to rate how important different types of training and training resources would be in helping health care professionals prescribe and interact with a digital health tool. In 16b, we ask how likely you believe HCPs would be to participate in different types of training. Assume the HCP will receive computerized reports with the output from the digital tool.

16a. Importance to HCP's ability to prescribe and interact with digital health tool. (Please rate each item on a scale from 1 to 9 with 1 = not at all important 5 = somewhat important 9 = extremely important)

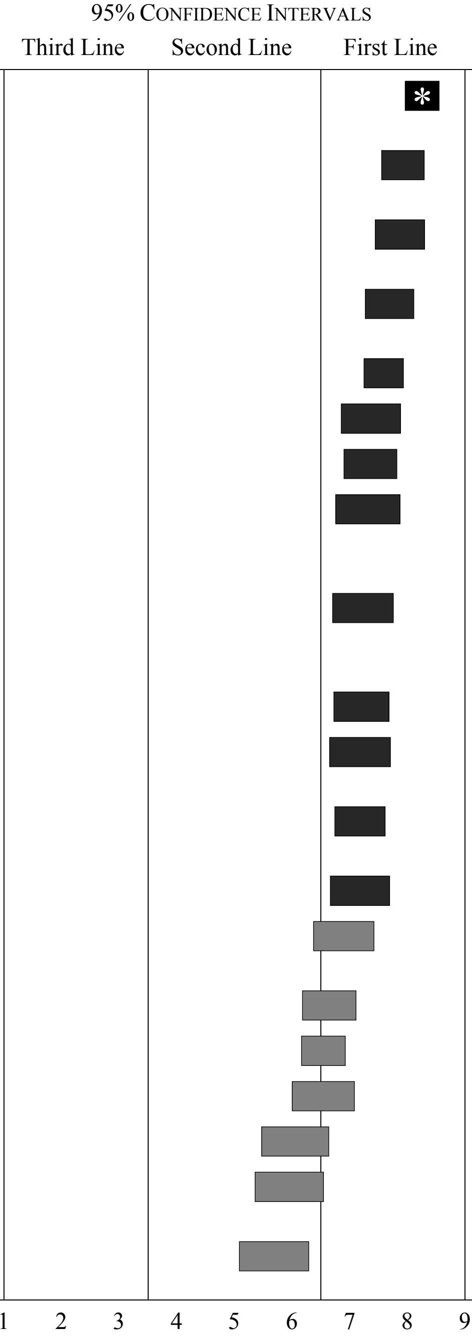

	N	Avg (SD)	*Tr of Chc* %	1st Line %	2nd Line %	3rd Line %
Clear rationale provided to HCPs about how using this device can improve outcomes	39	8.3 (0.9)	*54*	95	5	0
Provision of hands-on work with device/dashboard during training sessions	39	7.9 (1.2)	*46*	87	13	0
Inclusion of clinical examples and case materials as core elements of the training.	39	7.9 (1.4)	*44*	92	5	3
Clear and concise tutorial provided in the digital device	39	7.7 (1.3)	*33*	85	15	0
Technical call center support	39	7.6 (1.1)	*21*	90	10	0
Prepared handouts to give to patients	38	7.4 (1.6)	*32*	82	16	3
In-person training sessions	39	7.4 (1.5)	*28*	69	31	0
Simple platform that can be learned with user guide and video demonstration without requiring in-person training	38	7.3 (1.8)	*32*	74	21	5
Having the HCP use the digital system as a "patient" for a trial period to become familiar with its features	39	7.2 (1.7)	*28*	77	21	3
Complete protocol and user guide	39	7.2 (1.5)	*15*	79	15	5
Training provided in HCP's office (detailing approach)	39	7.2 (1.7)	*23*	64	31	5
Availability of follow-up training sessions (if needed)	39	7.2 (1.4)	*15*	72	28	0
CME credit for completing training	39	7.2 (1.6)	*28*	67	31	3
In-person training followed by web-based video reinforcement	39	6.9 (1.7)	*18*	64	33	3
Video-based online training module	39	6.6 (1.5)	*5*	67	28	5
Training webinars	39	6.5 (1.2)	*0*	69	28	3
FAQs/user forums on a website	39	6.5 (1.7)	*10*	56	36	8
Online chat support for questions/technical issues	39	6.1 (1.8)	*5*	46	46	8
Work with medical schools to provide training in digital health technology	39	5.9 (1.9)	*13*	38	49	13
Availability of consultation groups (e.g., groups involving other HCPs)	38	5.7 (1.9)	*8*	37	50	13

16b. Likelihood that HCP would participate in this type of training. (Please rate each item on a scale from 1 to 9 with 1 = not at all likely 5 = somewhat likely 9 = extremely likely)

	95% Confidence Intervals: Third Line	Second Line	First Line	*N*	Avg (SD)	*Tr of Chc*	1st Line	2nd Line	3rd Line
Clear and concise tutorial provided in the digital device				38	7.3 (1.7)	*24*	84	11	5
Individualized training provided in HCP's office				38	7.2 (1.7)	*29*	68	26	5
Technical call center support				38	6.4 (1.9)	*11*	55	39	5
Training webinars				38	6.0 (1.4)	*0*	47	47	5
Follow-up training sessions (if needed)				38	6.0 (1.7)	*0*	47	39	13
In-person training sessions				38	5.9 (1.9)	*13*	45	39	16
Having the HCP use the digital system as a "patient" for a trial period to become familiar with its features				38	5.9 (2.3)	*11*	47	34	18
Video-based online training module				38	5.8 (1.7)	*5*	37	53	11
Complete protocol and user guide				38	5.7 (1.9)	*8*	37	50	13
FAQs/user forums on a website				38	5.7 (2.1)	*3*	39	42	18
In-person training followed by web-based video reinforcement				38	5.6 (1.8)	*0*	34	50	16
Online chat support for questions/technical issues				38	5.2 (2.0)	*3*	29	47	24
Consultation groups (e.g., groups involving other HCPs)				38	4.8 (2.1)	*0*	29	34	37
	1 2 3	4 5 6	7 8 9			*%*	%	%	%

Question 17. **Provision of training and support to PATIENTS by HCPs**. Assume that the digital health tool designed for use with a patient with serious mental illness will be prescribed and monitored by an HCP. When we refer to HCPs here, we mean any professional providing health care services to patients with mental illnesses (e.g., psychiatrist, psychologist, nurse practitioner, clinical social worker). Thus, the HCP will be part of the system so that the design of the HCP's participation must be optimized. In the next two questions, we ask you to rate a number of different types of training and training resources for patients that would be provided by HCPs in terms of 17a) how important they would be in helping patients successfully engage with and use a digital health tool and then 17b) how difficult you believe each activity would be for the average HCP. Assume the HCP will receive computerized reports with the output from the digital tool.

17a. How IMPORTANT would each activity be in order to enable patients to successfully engage with and use a digital health tool? (Please rate each item on a scale from 1 to 9 with 1 = not at all important 5 = somewhat important 9 = extremely important)

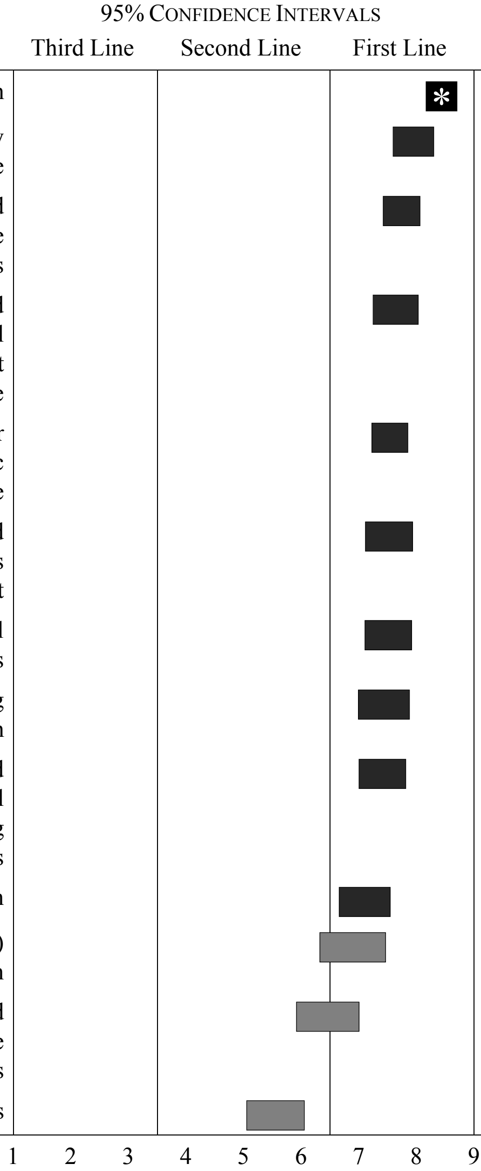

	N	Avg (SD)	*Tr of Chc*	1st Line	2nd Line	3rd Line
Initial training of patients on the system	39	8.4 (0.9)	*64*	97	3	0
Responding to technical problems identified by patients as quickly as possible	39	7.9 (1.1)	*41*	92	8	0
Verbalizing or otherwise providing specific and clear feedback about the data gathered via the digital health tools during treatment sessions	39	7.7 (1.0)	*26*	90	10	0
Providing reinforcement for patient's continued engagement with tools, independent of clinical information reported or outcomes within the first week of use	39	7.6 (1.2)	*26*	85	15	0
Arranging for treatment coordinator or other member of the treatment team to provide ad hoc support for system use	39	7.5 (1.0)	*18*	87	13	0
Developing ongoing reinforcement/reward systems for successful engagement with tools and/or successful self-management	38	7.5 (1.3)	*18*	84	13	3
Helping patients set goals and tasks on digital health tools	39	7.5 (1.3)	*28*	82	18	0
Identifying trends in digital data and responding immediately to critical clinical information	39	7.4 (1.4)	*26*	82	15	3
Providing reinforcement for patient's continued engagement with tools, independent of clinical information reported or outcomes on an ongoing basis	39	7.4 (1.3)	*23*	82	18	0
Follow-up/ongoing training on the system	39	7.1 (1.4)	*21*	69	31	0
Initial training of family caregivers (if applicable) on the system	38	6.9 (1.8)	*18*	63	34	3
Verbalizing or otherwise providing specific and clear feedback about the data gathered via the digital health tools between treatment sessions	39	6.5 (1.7)	*10*	54	41	5
Sharing information with family/caregivers	38	5.6 (1.6)	*0*	24	68	8
			%	%	%	%

17b. How DIFFICULT would each activity would be for the average HCP? (Please rate each item on a scale from 1 to 9 with 1 = extremely difficult 5 = somewhat difficult 9 = not at all difficultt)

95% CONFIDENCE INTERVALS

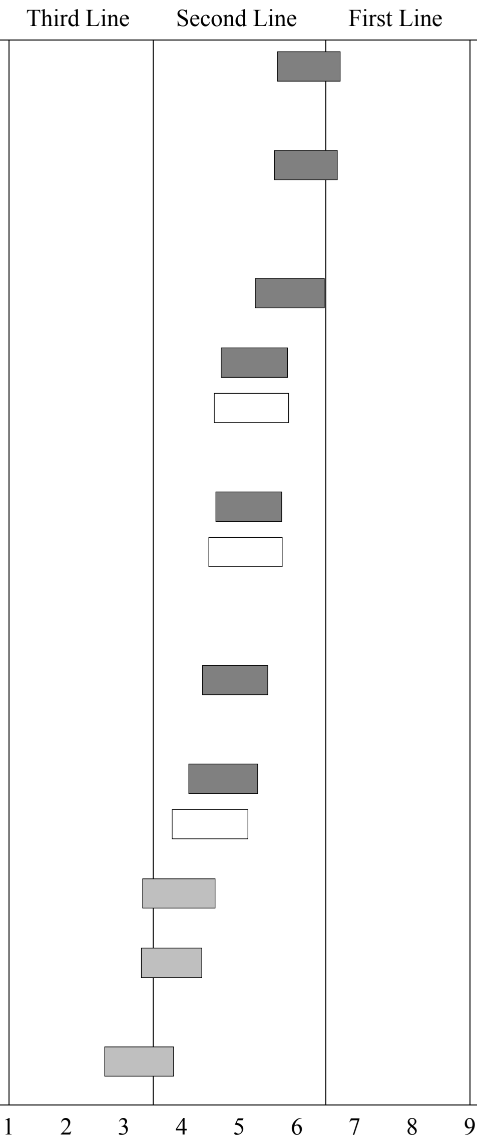

	N	Avg (SD)	*Tr of Chc* %	1st Line %	2nd Line %	3rd Line %
Verbalizing or otherwise providing specific and clear feedback about the data gathered via the digital health tools during treatment sessions	39	6.2 (1.7)	*5*	56	33	10
Providing reinforcement for patient's continued engagement with tools, independent of clinical information reported or outcomes within the first week of use	39	6.2 (1.7)	*8*	51	41	8
Helping patients set goals and tasks on digital health tools	39	5.9 (1.9)	*10*	41	41	18
Initial training of patients on the system	39	5.3 (1.8)	*8*	26	56	18
Arranging for treatment coordinator or other member of the treatment team to provide ad hoc support for system use	39	5.2 (2.1)	*5*	31	41	28
Follow-up/ongoing training on the system	38	5.2 (1.8)	*5*	21	63	16
Providing reinforcement for patient's continued engagement with tools, independent of clinical information reported or outcomes on an ongoing basis	39	5.1 (2.0)	*3*	36	41	23
Developing ongoing reinforcement/reward systems for successful engagement with tools and/or successful self-management	39	4.9 (1.8)	*3*	18	59	23
Sharing information with family/caregivers	39	4.7 (1.9)	*5*	15	54	31
Initial training of family caregivers (if applicable) on the system	39	4.5 (2.1)	*5*	18	41	41
Identifying trends in digital data and responding immediately to critical clinical information	38	3.9 (2.0)	*0*	13	50	37
Verbalizing or otherwise providing specific and clear feedback about the data gathered via the digital health tools between treatment sessions	39	3.8 (1.7)	*0*	10	36	54
Responding to technical problems identified by patients as quickly as possible	39	3.3 (1.9)	*0*	8	28	64

Question 19. Patient characteristics that affect outcomes in psychotherapy/psychosocial interventions. How much do you believe the following patient characteristics affect the chances of achieving favorable outcomes in psychotherapy/psychosocial interventions? Thinking about this question, please use your experience with all types of therapies/interventions and different types of patients. (Please rate each item on a scale from 1 to 9 with 1 = likely to have a very adverse effect on outcomes 5 = not likely to influence outcomes much one way or another 9 = extremely likely to promote good outcomes.)

19a. Patient Characteristics

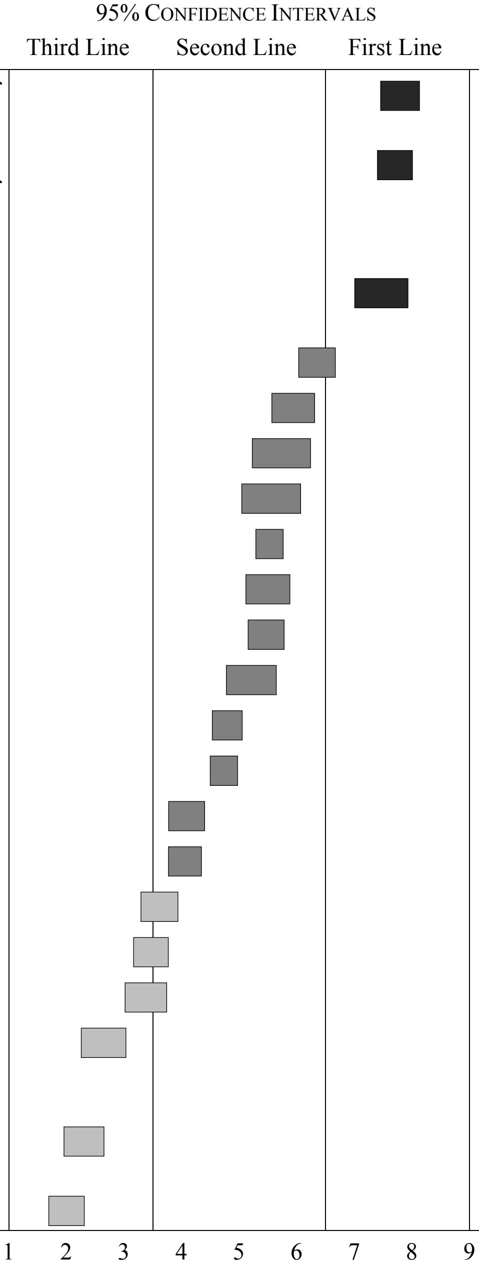

	N	Avg (SD)	*Tr of Chc*	1st Line	2nd Line	3rd Line
Positive expectations about therapy on the part of the patient	34	7.8 (1.0)	*29*	94	6	0
Resources that facilitate access to treatment (availability of transportation, availability of HCPs trained to provide psychotherapy, insurance coverage)	34	7.7 (0.9)	*21*	91	9	0
Positive social support (i.e., patient has an involved significant other or caregiver)	34	7.5 (1.4)	*21*	82	15	3
Bachelor's degree or more education	34	6.4 (0.9)	*0*	44	56	0
Currently married	33	5.9 (1.1)	*3*	33	67	0
18-44 years old	34	5.7 (1.5)	*3*	29	65	6
< 18 years old	34	5.6 (1.5)	*3*	26	65	9
High school or more education	34	5.5 (0.7)	*0*	9	91	0
45-64 years old	34	5.5 (1.1)	*0*	18	82	0
Female gender	34	5.5 (0.9)	*0*	12	85	3
65+ years old	33	5.2 (1.3)	*0*	12	79	9
Male gender	34	4.8 (0.8)	*0*	3	94	3
Minority status	34	4.7 (0.7)	*0*	3	94	3
Low socioeconomic status	34	4.1 (0.9)	*0*	0	79	21
Did not complete high school	34	4.1 (0.9)	*0*	0	74	26
Low health literacy	34	3.6 (1.0)	*0*	0	53	47
Low literacy (i.e., reading ability)/low numeracy	34	3.5 (0.9)	*0*	0	44	56
Low IQ	34	3.4 (1.1)	*0*	0	41	59
Severe psychosocial stressors (e.g., poverty, general medical problems, abusive relationship, legal problems)	34	2.6 (1.2)	*0*	0	18	82
Serious level of chaos and disorganization in the person's life/environment	33	2.3 (1.0)	*0*	0	6	94
Low motivation	33	2.0 (0.9)	*0*	0	6	94
			%	%	%	%

19b. Disorders, Signs, and Symptoms

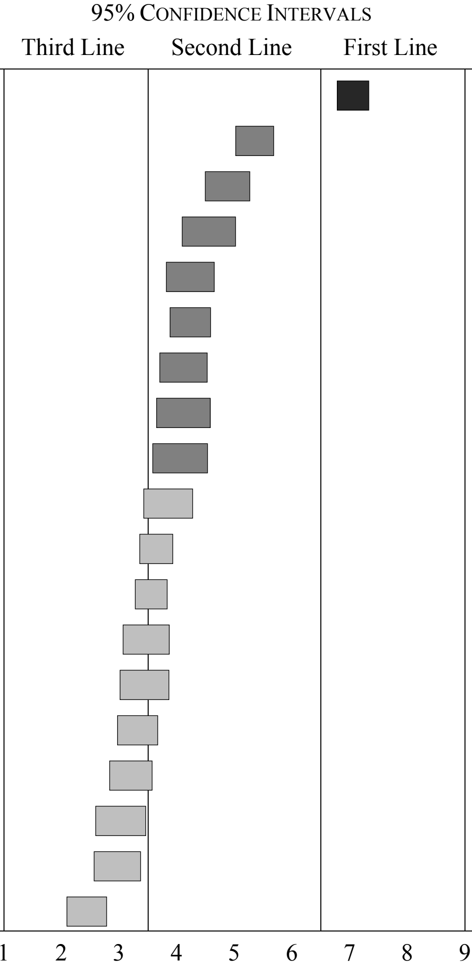

	N	Avg (SD)	*Tr of Chc*	1st Line	2nd Line	3rd Line
Good occupational functioning	34	7.1 (0.8)	*0*	76	24	0
Diagnosis of major depressive disorder	34	5.4 (1.0)	*0*	15	85	0
Diagnosis of bipolar disorder	34	4.9 (1.1)	*0*	9	82	9
Suicidal ideation	34	4.6 (1.4)	*0*	6	79	15
Diagnosis of schizophrenia	34	4.2 (1.2)	*0*	3	68	29
Sensitivity to overstimulation	34	4.2 (1.0)	*0*	3	74	24
Longer duration of illness	34	4.1 (1.2)	*0*	0	65	35
Non-suicidal self-injurious behaviors	34	4.1 (1.4)	*0*	3	62	35
Multiple previous episodes	33	4.1 (1.4)	*0*	3	70	27
Multiple comorbid Axis I disorders	34	3.9 (1.3)	*0*	3	62	35
Low frustration tolerance	34	3.6 (0.8)	*0*	0	56	44
Low energy/fatigue	34	3.6 (0.8)	*0*	0	47	53
Presence of Axis II comorbidity	34	3.5 (1.2)	*0*	0	50	50
Greater severity of delusions/paranoia	34	3.4 (1.3)	*0*	0	44	56
Aggression/agitation	34	3.3 (1.0)	*0*	0	44	56
Greater severity of neurocognitive impairment	34	3.2 (1.1)	*0*	0	41	59
Greater severity of negative symptoms	34	3.0 (1.3)	*0*	3	21	76
Greater severity of disorganization symptoms	34	3.0 (1.2)	*0*	3	18	79
Acute substance use	34	2.4 (1.0)	*0*	0	15	85
			%	%	%	%

19c. Appraisals and Patient Experience

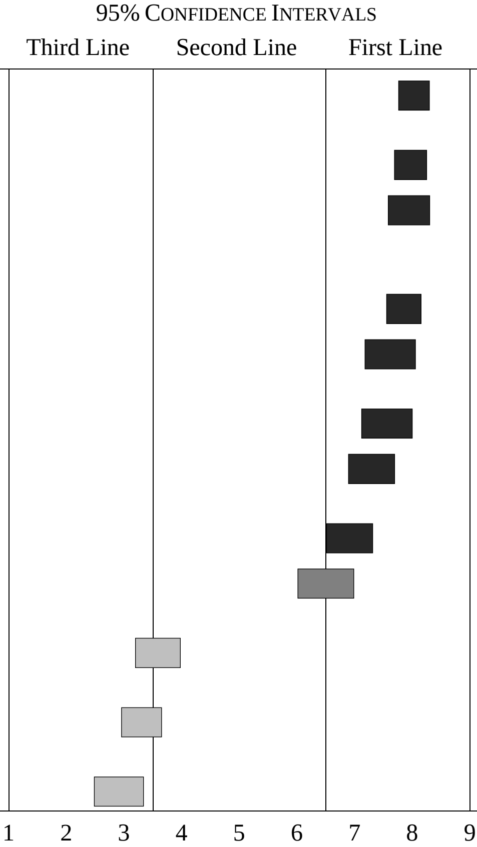

	N	Avg (SD)	*Tr of Chc*	1st Line	2nd Line	3rd Line
Positive bond with healthcare professional (good therapeutic alliance)	34	8.0 (0.8)	*32*	100	0	0
Perception of treatment as beneficial	34	8.0 (0.8)	*29*	97	3	0
Self-efficacy beliefs about ability to participate in and benefit from psychotherapy/psychosocial intervention	34	7.9 (1.1)	*35*	97	3	0
Agreement with tasks and goals of treatment	34	7.9 (0.9)	*29*	97	3	0
Willingness to complete tasks/homework between treatment sessions	34	7.6 (1.3)	*32*	82	18	0
Readiness to change	34	7.6 (1.3)	*26*	76	24	0
History of good adherence to medication and other treatment	34	7.3 (1.2)	*15*	85	15	0
Psychological mindedness	34	6.9 (1.2)	*12*	62	38	0
Engagement with mutual help organizations and/or peer support groups	34	6.5 (1.4)	*9*	47	50	3
Stigma/discomfort/social anxiety about seeking care for mental or behavioral problems	34	3.6 (1.2)	*0*	0	38	62
Negative past experience with psychotherapy/psychosocial intervention	33	3.3 (1.0)	*0*	0	33	67
Limited insight into disorder	34	2.9 (1.3)	*0*	0	32	68
			%	%	%	%
