# Supplementary material for: Expert Consensus Survey on Digital Health Tools for Patients With Serious Mental Illness: Optimizing for User Characteristics and User Support
Source: JMIR Ment Health. 2018 Jun 12;5(2):e46. doi: 10.2196/mental.9777 (PMC6019847; doi:10.2196/mental.9777)
Supplement: Multimedia Appendix 2 [file mental_v5i2e46_app2.pdf]

Question 1. Characteristics of PATIENTS that affect ability to engage with and use a digital health tool How much do you believe the following patient characteristics affect the chances that a patient can successfully engage with and use a digital health tool? In answering this and subsequent questions, please consider the type of digital health tool(s) with which you have the most experience.

Please rate each item on a scale from 1 to 9 with: 1 = likely to make it very difficult for person to engage with and use 5 = not likely to influence ability to engage with or use much one way or another 9 = extremely likely to promote engagement and ability to use.

1a. Patient Characteristics

	N	*"1"*	*"2"*	*"3"*	*"4"*	*"5"*	*"6"*	*"7"*	*"8"*	*"9"*	*Avg*	*SD*	*Chc*	*1st*	*2nd*	*3rd*	*CI_lo*	*CI_hi*	*Rank*	*Consensus*
Interest in using state-of-the-art technology	39	1	0	0	0	0	3	7	7	21	8.0	1.5	54%	90%	8%	3%	7.5	8.5	Chc	Yes
Resources that facilitate access to treatment (availability of Wi-Fi or data plan, applicable hardware, insurance coverage)	40	0	0	0	0	1	2	11	16	10	7.8	1.0	25%	93%	8%	0%	7.5	8.1	1st	Yes
Positive expectations on the part of the patient about using a digital health tool	40	0	0	0	0	1	3	11	14	11	7.8	1.0	28%	90%	10%	0%	7.5	8.1	1st	Yes
Owns and uses a smart phone/computer/tablet	40	0	0	0	0	0	4	11	16	9	7.8	0.9	23%	90%	10%	0%	7.5	8.0	1st	Yes
Positive social support (i.e., patient has an involved significant other or caregiver)	40	0	0	0	0	1	3	21	11	4	7.4	0.9	10%	90%	10%	0%	7.1	7.6	1st	Yes
< 18 years old	40	0	0	2	2	3	2	10	8	13	7.3	1.8	33%	78%	18%	5%	6.8	7.8	1st	Yes
18-44 years old	40	0	0	0	0	4	7	11	11	7	7.3	1.2	18%	73%	28%	0%	6.9	7.6	1st	Yes
Bachelor’s degree or more education	40	0	0	0	0	3	9	16	10	2	7.0	1.0	5%	70%	30%	0%	6.7	7.3	1st	Yes
High school or more education	40	0	0	1	1	10	11	14	3	0	6.1	1.1	0%	43%	55%	3%	5.8	6.5	2nd	Yes
Female gender	40	0	0	0	0	28	3	6	2	1	5.6	1.1	3%	23%	78%	0%	5.3	6.0	2nd	Yes
45-64 years old	40	0	0	1	6	14	8	10	1	0	5.6	1.2	0%	28%	70%	3%	5.2	5.9	2nd	Yes
Currently married	40	0	0	0	0	27	7	4	2	0	5.5	0.9	0%	15%	85%	0%	5.3	5.8	2nd	Yes
Male gender	40	0	0	2	1	25	3	7	1	1	5.5	1.2	3%	23%	73%	5%	5.1	5.8	2nd	Yes
Minority status	40	0	1	3	5	26	3	2	0	0	4.8	1.0	0%	5%	85%	10%	4.5	5.1	2nd	Yes
Low socioeconomic status	40	2	0	12	14	10	0	2	0	0	4.0	1.2	0%	5%	60%	35%	3.6	4.3	2nd	Yes
Did not complete high school	40	1	2	12	15	9	1	0	0	0	3.8	1.0	0%	0%	63%	38%	3.5	4.1	3rd	Yes
65+ years old	40	1	8	12	7	6	4	2	0	0	3.7	1.5	0%	5%	43%	53%	3.3	4.2	3rd	Yes
Low health literacy	40	1	7	15	8	9	0	0	0	0	3.4	1.1	0%	0%	43%	58%	3.1	3.8	3rd	Yes
Severe psychosocial stressors (e.g., poverty, general medical problems, abusive relationship, legal problems)	40	4	12	13	8	2	1	0	0	0	2.9	1.2	0%	0%	28%	73%	2.5	3.2	3rd	Yes
Low IQ	40	3	7	23	7	0	0	0	0	0	2.9	0.8	0%	0%	18%	83%	2.6	3.1	3rd	Yes
None or minimal knowledge/ability/comfort using technology (e.g., computers, tablets, mobile phones)	39	1	16	16	4	2	0	0	0	0	2.7	0.9	0%	0%	15%	85%	2.5	3.0	3rd	Yes
Low literacy (i.e., reading ability)/numeracy	40	5	16	17	1	1	0	0	0	0	2.4	0.8	0%	0%	5%	95%	2.2	2.7	3rd	Yes
Low motivation	40	10	16	12	2	0	0	0	0	0	2.2	0.9	0%	0%	5%	95%	1.9	2.4	3rd	Yes
Serious level of chaos and disorganization in the person’s life/environment	40	12	16	9	3	0	0	0	0	0	2.1	0.9	0%	0%	8%	93%	1.8	2.4	3rd	Yes

1b. Disorders, Signs, and Symptoms

	N	"1"	"2"	"3"	"4"	"5"	"6"	"7"	"8"	"9"	Avg	SD	Chc	1st	2nd	3rd	CI_lo	CI_hi	Rank	Consensus
Good occupational functioning	39	0	0	0	1	3	6	17	11	1	6.9	1.1	3%	74%	26%	0%	6.6	7.3	1st	Yes
Diagnosis of bipolar disorder	40	0	0	2	5	25	4	2	1	1	5.2	1.1	3%	10%	85%	5%	4.8	5.5	2nd	Yes
Diagnosis of major depressive disorder	40	0	0	4	6	23	2	3	1	1	5.0	1.3	3%	13%	78%	10%	4.6	5.4	2nd	Yes
Non-suicidal self-injurious behaviors	40	0	0	0	7	29	3	1	0	0	5.0	0.6	0%	3%	98%	0%	4.8	5.1	2nd	Yes
Suicidal ideation	40	0	3	4	3	26	3	0	1	0	4.7	1.2	0%	3%	80%	18%	4.3	5.0	2nd	Yes
Multiple previous episodes	40	1	1	3	7	25	3	0	0	0	4.6	1.0	0%	0%	88%	13%	4.3	4.9	2nd	Yes
Diagnosis of schizophrenia	40	0	3	5	7	20	3	2	0	0	4.5	1.2	0%	5%	75%	20%	4.2	4.9	2nd	Yes
Longer duration of illness	40	0	4	5	9	15	5	2	0	0	4.5	1.3	0%	5%	73%	23%	4.0	4.9	2nd	Yes
Sensitivity to overstimulation	40	0	4	8	10	16	0	1	1	0	4.2	1.3	0%	5%	65%	30%	3.8	4.6	2nd	Yes
Presence of Axis II comorbidity	40	1	3	6	10	20	0	0	0	0	4.1	1.1	0%	0%	75%	25%	3.8	4.5	2nd	Yes
Multiple comorbid Axis I disorders	40	1	4	11	6	18	0	0	0	0	3.9	1.2	0%	0%	60%	40%	3.5	4.3	2nd	Yes
Greater severity of delusions/paranoia	40	2	7	10	9	10	2	0	0	0	3.6	1.3	0%	0%	53%	48%	3.2	4.0	3rd	Yes
Aggression/agitation	40	4	4	10	11	11	0	0	0	0	3.5	1.3	0%	0%	55%	45%	3.1	3.9	3rd	Yes
Acute substance use	39	4	7	9	11	7	1	0	0	0	3.3	1.3	0%	0%	49%	51%	2.9	3.7	3rd	Yes
Low energy/fatigue	40	2	4	16	16	2	0	0	0	0	3.3	0.9	0%	0%	45%	55%	3.0	3.6	3rd	Yes
Low frustration tolerance	40	2	8	19	9	2	0	0	0	0	3.0	0.9	0%	0%	28%	73%	2.7	3.3	3rd	Yes
Greater severity of negative symptoms	39	6	12	9	4	7	1	0	0	0	2.9	1.4	0%	0%	31%	69%	2.5	3.4	3rd	Yes
Greater severity of disorganization symptoms	40	4	14	11	8	2	0	1	0	0	2.9	1.3	0%	3%	25%	73%	2.5	3.2	3rd	Yes
Greater severity of neurocognitive impairment	40	6	12	11	8	2	1	0	0	0	2.8	1.2	0%	0%	28%	73%	2.4	3.2	3rd	Yes

1c. Appraisals and Patient Experience

	N	"1"	"2"	"3"	"4"	"5"	"6"	"7"	"8"	"9"	Avg	SD	Chc	1st	2nd	3rd	CI_lo	CI_hi	Rank	Consensus
Perception of digital treatment as beneficial	39	0	0	0	0	0	0	7	19	13	8.2	0.7	33%	100%	0%	0%	7.9	8.4	1st	Yes
Agreement with tasks and goals of digital treatment	40	0	0	0	0	0	1	12	16	11	7.9	0.8	28%	98%	3%	0%	7.7	8.2	1st	Yes
Self-efficacy beliefs about being able to use/operate the device	40	0	0	0	0	0	4	8	17	11	7.9	0.9	28%	90%	10%	0%	7.6	8.2	1st	Yes
Positive bond with healthcare professional (good therapeutic alliance)	40	0	0	0	0	0	4	12	17	7	7.7	0.9	18%	90%	10%	0%	7.4	8.0	1st	Yes
Willingness to complete tasks/homework between treatment sessions	39	0	0	0	0	0	4	12	16	7	7.7	0.9	18%	90%	10%	0%	7.4	7.9	1st	Yes
History of good adherence to medication and other treatment	40	0	0	0	0	0	6	17	11	6	7.4	0.9	15%	85%	15%	0%	7.1	7.7	1st	Yes
Readiness to change	40	0	0	0	0	2	7	12	14	5	7.3	1.1	13%	78%	23%	0%	7.0	7.7	1st	Yes
Psychological mindedness	40	0	0	0	0	5	10	16	9	0	6.7	1.0	0%	63%	38%	0%	6.4	7.0	2nd	Yes
Engagement with mutual help organizations and/or peer support groups	40	0	0	0	1	6	13	10	8	2	6.6	1.2	5%	50%	50%	0%	6.2	7.0	2nd	Yes
Stigma/discomfort/social anxiety about seeking face-to-face care for mental or behavioral problems	40	1	0	3	1	9	12	10	2	2	5.9	1.6	5%	35%	55%	10%	5.4	6.4	2nd	Yes
Negative past experience with treatment	40	4	3	12	11	7	3	0	0	0	3.6	1.4	0%	0%	53%	48%	3.2	4.0	3rd	Yes
Limited insight into disorder	40	3	8	12	8	8	1	0	0	0	3.3	1.3	0%	0%	43%	58%	2.9	3.7	3rd	Yes

Question 2. Characteristics and Resources of HEALTH CARE PROFESSIONALS (HCPs) How helpful do you believe the following characteristics and resources are in enabling HCPs to successfully incorporate digital health tools in their practices?

Please rate each item on a scale from 1 to 9 with 1 = not at all helpful 5 = somewhat helpful 9 = extremely helpful

	N	*"1"*	*"2"*	*"3"*	*"4"*	*"5"*	*"6"*	*"7"*	*"8"*	*"9"*	*Avg*	*SD*	*Chc*	*1st*	*2nd*	*3rd*	*CI_lo*	*CI_hi*	*Rank*	*Consensus*
HCP enthusiastic about/willing to work with patients using digital health tools	40	0	0	0	0	0	0	2	16	22	8.5	0.6	55%	100%	0%	0%	8.3	8.7	Chc	Yes
Availability of staff to support the ongoing use of the prescribed technology (e.g. make follow-up phone calls, monitor progress)	40	0	0	0	1	0	1	4	14	20	8.3	1.0	50%	95%	5%	0%	7.9	8.6	Chc	Yes
Availability of necessary equipment (e.g. computers, broadband internet if needed, mobile devices) for delivering patient treatment on a regular basis	40	0	0	0	0	2	0	5	13	20	8.2	1.0	50%	95%	5%	0%	7.9	8.5	Chc	Yes
Availability of staff with technology skills to teach patients how to use technology initially	40	0	0	0	1	0	1	8	14	16	8.1	1.1	40%	95%	5%	0%	7.7	8.4	1st	Yes
Availability of necessary equipment (e.g. computers, broadband internet if needed, mobile devices) for patient use in the office for training	40	0	0	0	0	1	3	6	14	16	8.0	1.0	40%	90%	10%	0%	7.7	8.4	1st	Yes
HCP is experienced in using computers in practice	40	0	0	0	0	0	6	14	10	10	7.6	1.0	25%	85%	15%	0%	7.3	7.9	1st	Yes
HCP has familiarity with smart phone technology	39	0	0	0	0	0	6	14	9	10	7.6	1.0	26%	85%	15%	0%	7.3	7.9	1st	Yes
Availability of free trial version of tools	40	0	0	0	1	0	4	12	18	5	7.5	1.0	13%	88%	13%	0%	7.2	7.8	1st	Yes
Availability of a digital health tool that is consistent with the HCP's preferred theoretical orientation (e.g., a digital CBT tool for a therapist trained in CBT)	40	0	0	0	1	2	5	8	17	7	7.5	1.2	18%	80%	20%	0%	7.1	7.9	1st	Yes
Availability of 24/7 call center to provide technical support for HCP and patients	40	0	0	1	1	3	3	9	14	9	7.4	1.5	23%	80%	18%	3%	6.9	7.9	1st	Yes
HCP is ≤ 40 years old	40	1	1	1	1	7	6	14	5	4	6.4	1.8	10%	58%	35%	8%	5.8	6.9	2nd	Yes

Question 4. Potential benefits of using a digital health tool for PATIENTS. Assume the items below have been demonstrated to be benefits of using a digital health tool for patients with psychiatric disorders. For each, please rate how likely you believe it would be to motivate a patient to use the digital health tool.

Please rate each item on a scale from 1 to 9 with 1 = not at all likely to motivate patient 5 = somewhat likely to motivate patient 9 = extremely likely to motivate patient

	N	*"1"*	*"2"*	*"3"*	*"4"*	*"5"*	*"6"*	*"7"*	*"8"*	*"9"*	*Avg*	*SD*	*Chc*	*1st*	*2nd*	*3rd*	*CI_lo*	*CI_hi*	*Rank*	*Consensus*
Improved functioning (e.g., social and work functioning)	40	0	1	0	0	0	2	8	14	15	7.9	1.3	38%	93%	5%	3%	7.5	8.3	1st	Yes
Reduced symptomatology	40	0	0	0	1	0	6	4	17	12	7.8	1.2	30%	83%	18%	0%	7.4	8.2	1st	Yes
Receiving feedback/support from clinicians via the digital health system between face-to-face sessions	39	0	0	0	0	2	4	9	16	8	7.6	1.1	21%	85%	15%	0%	7.3	8.0	1st	Yes
Ability to engage with HCP periodically after discharge from face-to-face sessions	40	0	0	0	0	2	7	6	18	7	7.5	1.1	18%	78%	23%	0%	7.2	7.9	1st	Yes
Increased interaction with treatment team via digital health device in geographic areas where face-to-face access to HCPs is limited	40	0	1	0	0	2	6	10	11	10	7.4	1.5	25%	78%	20%	3%	6.9	7.9	1st	Yes
Increased confidence/self-efficacy and hope related to his or her health care	40	0	0	1	0	0	9	12	9	9	7.4	1.3	23%	75%	23%	3%	6.9	7.8	1st	Yes
Reduction in number of hospitalizations	38	1	1	1	0	3	3	12	7	10	7.1	1.9	26%	76%	16%	8%	6.5	7.7	2nd	Yes
Elimination or reduction of problems with transportation to treatment	40	0	0	0	1	4	8	11	12	4	7.0	1.3	10%	68%	33%	0%	6.6	7.4	1st	Yes
More personalized/tailored treatment approach can be offered by technology	40	0	0	0	2	4	6	10	17	1	7.0	1.3	3%	70%	30%	0%	6.6	7.4	1st	Yes
Increased social engagement enabled by technology	40	1	0	0	0	4	7	15	6	7	7.0	1.5	18%	70%	28%	3%	6.5	7.5	2nd	Yes
Receiving prompt helpful automated feedback in response to input/questions	40	0	1	0	1	3	7	12	12	4	7.0	1.4	10%	70%	28%	3%	6.5	7.4	1st	Yes
Increased willingness to engage in care for those who are reluctant to meet face-to-face with HCPs	40	1	0	1	3	2	8	11	9	5	6.7	1.8	13%	63%	33%	5%	6.2	7.2	2nd	Yes
Access to state-of the art treatment tools	40	0	1	0	0	7	12	8	8	4	6.6	1.5	10%	50%	48%	3%	6.2	7.1	2nd	Yes
Better self-understanding of thinking, feelings, and behaviors	40	1	0	0	2	3	10	13	9	2	6.6	1.5	5%	60%	38%	3%	6.2	7.1	2nd	Yes
Decreased stigma related to seeking mental health care	40	1	0	1	3	5	6	13	7	4	6.5	1.7	10%	60%	35%	5%	6.0	7.0	2nd	Yes
Improved communication/understanding between patients and family members because of family integration in digital system	39	1	0	2	1	4	10	10	8	3	6.5	1.7	8%	54%	38%	8%	5.9	7.0	2nd	Yes
Self-management and ability to record personal information (e.g., about adherence, symptoms)	40	0	1	2	1	4	14	7	8	3	6.4	1.6	8%	45%	48%	8%	5.9	6.9	2nd	Yes
Around the clock access to a digital health tool that is part of the patient's treatment plan	39	0	1	0	0	9	11	9	8	1	6.4	1.3	3%	46%	51%	3%	6.0	6.8	2nd	Yes
Digital links to wide range of resources and support	40	1	1	0	1	9	7	13	8	0	6.2	1.6	0%	53%	43%	5%	5.7	6.7	2nd	Yes
Increased understanding and investment in treatment plan	40	1	0	2	3	6	10	11	4	3	6.1	1.7	8%	45%	48%	8%	5.6	6.7	2nd	Yes
Ability of multiple HCPs involved in patient's care (e.g., case manager, psychologist) to access the data	40	0	0	3	7	5	8	9	5	3	6.0	1.7	8%	43%	50%	8%	5.5	6.5	2nd	Yes
Improved patient adherence to medication and treatment plan	39	0	2	4	3	5	10	10	5	0	5.7	1.7	0%	38%	46%	15%	5.2	6.3	2nd	Yes
Potential to reduce utilization of health care services and therefore cost of treatment	40	2	2	4	1	11	4	9	5	2	5.6	2.1	5%	40%	40%	20%	4.9	6.2	2nd	No
Media representation more engaging than face-to-face interactions	40	0	4	4	2	14	5	5	6	0	5.3	1.8	0%	28%	53%	20%	4.7	5.8	2nd	Yes

Question 5. Potential barriers/unintended consequences when using digital health tool (e.g., mobile health application, computerized treatment tool, website). Considering average patient with serious mental illness (schizophrenia, bipolar, or major depressive disorder), how likely do you believe each of the items below is to be a potential barrier and/or to be or lead to an unintended consequence for PATIENTS using a digital health tool?

Please rate each item on a scale from 1 to 9 with 1 = significant potential to be a barrier and/or an unintended consequence 5 = some potential to be a barrier and/or an unintended consequence 9 = minimal potential to be a barrier and/or an unintended consequence

	N	*"1"*	*"2"*	*"3"*	*"4"*	*"5"*	*"6"*	*"7"*	*"8"*	*"9"*	*Avg*	*SD*	*Chc*	*1st*	*2nd*	*3rd*	*CI_lo*	*CI_hi*	*Rank*	*Consensus*
Increase in family conflict due to information provided by the digital tool	39	2	4	4	6	2	3	8	7	3	5.5	2.4	8%	46%	28%	26%	4.7	6.2	2nd	No
Depersonalization of patient care and potential damage to the therapeutic relationship	40	4	3	7	3	3	6	8	3	3	5.0	2.5	8%	35%	30%	35%	4.3	5.8	2nd	No
Patient's use of digital health tool in isolation leads to decreased face-to-face interaction with clinician and/or treatment team	40	5	3	5	3	8	4	6	6	0	4.8	2.3	0%	30%	38%	33%	4.1	5.5	2nd	No
Patient sets unrealistic goal and becomes discouraged	39	4	6	9	5	2	1	5	6	1	4.4	2.5	3%	31%	21%	49%	3.6	5.2	2nd	Yes
Patient disappointed by not receiving prompt response from HCP	40	0	6	12	7	3	4	6	2	0	4.3	1.9	0%	20%	35%	45%	3.7	4.9	2nd	No
Patient feels frustrated and discouraged with using the technology	40	10	6	9	3	3	3	3	2	1	3.5	2.3	3%	15%	23%	63%	2.8	4.2	3rd	Yes
Patient has concerns about privacy	40	6	10	8	6	4	3	1	2	0	3.4	1.9	0%	8%	33%	60%	2.8	4.0	3rd	Yes
Patient finds the digital health tool intrusive	40	8	11	10	2	2	1	4	2	0	3.2	2.1	0%	15%	13%	73%	2.5	3.9	3rd	Yes
Patient has concerns about being monitored or policed	40	8	12	7	6	2	2	2	0	1	3.1	1.9	3%	8%	25%	68%	2.5	3.6	3rd	Yes
Patient does not understand how to use the digital health tool	40	12	10	7	4	3	2	1	0	1	2.8	1.9	3%	5%	23%	73%	2.2	3.4	3rd	Yes
Patient finds it a burden to use the digital health tool	40	12	12	9	2	2	0	1	1	1	2.6	1.9	3%	8%	10%	83%	2.0	3.2	3rd	Yes
Patient does not believe that the intervention is well suited to his/her particular problem(s).	40	10	15	8	3	1	0	2	0	1	2.6	1.8	3%	8%	10%	83%	2.0	3.2	3rd	Yes
For following 2 items, consider only patients with psychosis:																				
Possible exacerbation of paranoid symptoms related to being monitored or "controlled"	40	9	2	3	4	4	3	6	8	1	4.8	2.7	3%	38%	28%	35%	3.9	5.6	2nd	No
Patient misinterprets interactions with the digital health tool because of paranoid delusions	40	7	4	5	3	4	3	5	8	1	4.7	2.7	3%	35%	25%	40%	3.9	5.5	2nd	No

Question 6. Potential benefits of using a digital health tool for HEALTH CARE PROFESSIONALS. Assume the items below have been demonstrated to be benefits of using a digital health tool for HCPs who treat patients with psychiatric disorders. For each, please rate how likely you believe it would be to motivate an HCP to use the digital health tool.

Please rate each item on a scale from 1 to 9 with 1 = not at all likely to motivate HCP 5 = somewhat likely to motivate HCP 9 = extremely likely to motivate HCP

	N	***"1"***	***"2"***	***"3"***	***"4"***	***"5"***	***"6"***	***"7"***	***"8"***	***"9"***	***Avg***	***SD***	***Chc***	***1st***	***2nd***	***3rd***	***CI_lo***	***CI_hi***	***Rank***	***Consensus***
Reimbursement by payers for time spent providing training to patients and family members about the digital health tool	39	0	0	0	0	1	1	1	12	24	8.5	0.9	62%	95%	5%	0%	8.2	8.7	Chc	Yes
Reimbursement by payers for time spent using/reviewing data from the digital health tool	40	0	1	0	0	1	1	0	11	26	8.4	1.3	65%	93%	5%	3%	8.0	8.8	Chc	Yes
Improved patient adherence to medication and treatment plan	40	0	0	1	0	0	2	8	14	15	8.0	1.2	38%	93%	5%	3%	7.6	8.3	1st	Yes
Improved patient functioning (e.g., social and work functioning)	39	0	0	0	0	0	4	9	12	14	7.9	1.0	36%	90%	10%	0%	7.6	8.2	1st	Yes
Reduced patient symptomatology	39	0	0	0	0	1	2	9	14	13	7.9	1.0	33%	92%	8%	0%	7.6	8.2	1st	Yes
Increased efficiency of care provision by freeing up HCP's time without compromising quality of care (i.e., technology as a treatment extender to reduce HCP burden)	39	0	1	0	0	1	3	9	10	15	7.8	1.4	38%	87%	10%	3%	7.3	8.2	1st	Yes
Increased ability to deliver evidence-based treatments that would not otherwise be delivered because of limited staff resources, limited trained staff, or cost barriers	40	0	0	0	0	3	5	7	17	8	7.6	1.2	20%	80%	20%	0%	7.2	7.9	1st	Yes
More accurate data about medication adherence	40	0	0	1	1	1	3	9	17	8	7.5	1.3	20%	85%	13%	3%	7.1	7.9	1st	Yes
Digital health tool provides input to common electronic health record (EHR) systems already in use	39	0	0	0	0	5	1	13	10	10	7.5	1.3	26%	85%	15%	0%	7.1	7.9	1st	Yes
Published evidence concerning efficacy of the digital health tool in improving patient outcomes	38	0	0	0	0	4	5	7	14	8	7.4	1.3	21%	76%	24%	0%	7.0	7.9	1st	Yes
Replacing routine assessment and monitoring functions, thus increasing availability of HCP for more complex cases	40	0	2	0	0	0	4	14	9	11	7.4	1.6	28%	85%	10%	5%	6.9	7.9	1st	Yes
Reduction in number of hospitalizations	40	0	1	0	1	3	7	10	8	10	7.2	1.6	25%	70%	28%	3%	6.7	7.7	1st	Yes
Potential to achieve improved patient outcomes by being able to make more evidence-based treatment decisions	40	0	0	0	0	5	5	15	8	7	7.2	1.2	18%	75%	25%	0%	6.8	7.6	1st	Yes
Improved accuracy of information for clinical decision-making based on objective momentary data about patient symptoms	39	0	0	0	2	4	6	9	10	8	7.2	1.5	21%	69%	31%	0%	6.7	7.6	1st	Yes
Access to more patient information (e.g., about adherence, symptoms)	40	0	0	0	0	2	10	14	9	5	7.1	1.1	13%	70%	30%	0%	6.8	7.5	1st	Yes
Ability to provide state-of-the art treatment tools	40	0	1	0	1	3	7	13	10	5	7.0	1.5	13%	70%	28%	3%	6.5	7.4	1st	Yes
Ability of multiple HCPs involved in patient's care (e.g., case manager, psychologist) to access the data	40	0	1	0	1	3	10	9	10	6	7.0	1.5	15%	63%	35%	3%	6.5	7.4	2nd	Yes
Increased ability to personalize/tailor the treatment approach to the specific patient	40	0	1	0	1	3	12	7	10	6	6.9	1.5	15%	58%	40%	3%	6.4	7.4	2nd	Yes
Around the clock capability to assess and intervene with at-risk patients (e.g., with suicidal risk or increased agitation/aggression) in a way that may increase safety	40	0	1	2	2	6	4	7	8	10	6.8	2.0	25%	63%	30%	8%	6.2	7.4	2nd	Yes
Facilitation of patient disclosure about topics that patients may be reluctant to share in traditional face-to-face settings	40	0	1	1	0	4	8	13	11	2	6.8	1.4	5%	65%	30%	5%	6.3	7.2	2nd	Yes
Improved accuracy of information for clinical decision-making based on objective sensor data (e.g., external biometric sensors, GPS, Bluetooth)	40	0	0	0	4	5	7	12	8	4	6.7	1.5	10%	60%	40%	0%	6.2	7.1	2nd	Yes
Increased patient social engagement enabled by technology	39	0	0	1	1	6	9	10	10	2	6.6	1.4	5%	56%	41%	3%	6.2	7.1	2nd	Yes
Around the clock capability to to identify potential problems/early warning signs of acute episodes in order to intervene more quickly	40	0	1	2	3	4	7	7	10	6	6.6	1.9	15%	58%	35%	8%	6.0	7.2	2nd	Yes
Potential to reduce utilization of health care services (e.g., emergency, outpatient) and therefore cost of treatment	39	0	1	2	0	4	9	13	7	3	6.6	1.6	8%	59%	33%	8%	6.1	7.1	2nd	Yes
Better understanding of patient's thinking, feelings, and behaviors	39	0	1	1	0	4	7	19	7	0	6.6	1.3	0%	67%	28%	5%	6.2	7.0	2nd	Yes
Increased confidence/self-efficacy related to the patient's health care	40	0	1	1	2	5	9	12	8	2	6.5	1.5	5%	55%	40%	5%	6.0	6.9	2nd	Yes
Improved communication/understanding between patients and family members because of family integration in digital system (assuming this is a goal of treatment)	40	0	1	0	2	5	11	15	4	2	6.4	1.4	5%	53%	45%	3%	6.0	6.8	2nd	Yes

Opportunity to provide feedback/support to patients between face-to-face sessions	40	1	0	2	1	6	9	10	10	1	6.4	1.7	3%	53%	40%	8%	5.8	6.9	2nd	Yes
Ability to engage periodically with patients after discharge from face-to-face sessions	39	0	1	2	2	4	13	9	5	3	6.3	1.6	8%	44%	49%	8%	5.7	6.8	2nd	Yes

Question 7. Potential barriers and/or unintended consequences for HEALTH CARE PROFESSIONALS. Listed below are some potential barriers to and unintended consequences of HCPs' use of a digital health tool with patients with psychiatric disorders. How likely do you believe each of the items is to be a potential barrier and/or to be or lead to an unintended consequence for HCPs using a digital health tool with their patients?

Please rate each item on a scale from 1 to 9 with 1 = significant potential to be a barrier and/or an unintended consequence for an HCP 5 = some potential to be a barrier and/or an unintended consequence for an HCP 9 = minimal potential to be a barrier and/or an unintended consequence for an HCP

7a. Concerns about process and credibility of intervention

	N	*"1"*	*"2"*	*"3"*	*"4"*	*"5"*	*"6"*	*"7"*	*"8"*	*"9"*	*Avg*	*SD*	*Chc*	*1st*	*2nd*	*3rd*	*CI_lo*	*CI_hi*	*Rank*	*Consensus*
Loss of expert role due to inadequate technological knowledge	40	2	3	9	8	2	7	5	3	1	4.7	2.1	3%	23%	43%	35%	4.0	5.3	2nd	No
Concern about validation of measures	40	3	2	8	10	5	5	4	2	1	4.5	2.0	3%	18%	50%	33%	3.9	5.1	2nd	Yes
Concern about preserving the validity of evidence-based psychosocial interventions when delivered digitally	40	4	7	6	5	5	2	6	4	1	4.4	2.4	3%	28%	30%	43%	3.7	5.1	2nd	No
Uncertainty about how to discuss and explain the digital health tool to patients	40	6	5	9	3	4	2	1	9	1	4.4	2.6	3%	28%	23%	50%	3.6	5.2	2nd	Yes
Potential for blurring of patient-professional boundaries (e.g., digital tools allow contact outside scheduled face-to-face appointments)	40	3	4	12	5	4	3	3	6	0	4.4	2.2	0%	23%	30%	48%	3.7	5.0	2nd	No
Lack of centralized regulation/approval/rating body for novel technologies leads to unclear expected benefits	40	2	6	9	7	5	4	5	1	1	4.3	2.0	3%	18%	40%	43%	3.6	4.9	2nd	No
Inadequate information about how the processes delivered by digital systems can be integrated with in-person, usual care	40	6	15	9	1	4	2	3	0	0	3.0	1.8	0%	8%	18%	75%	2.5	3.5	3rd	Yes

7b. Concerns about usability/feasibility

	N	*"1"*	*"2"*	*"3"*	*"4"*	*"5"*	*"6"*	*"7"*	*"8"*	*"9"*	*Avg*	*SD*	*Chc*	*1st*	*2nd*	*3rd*	*CI_lo*	*CI_hi*	*Rank*	*Consensus*
Concern that the tool has not gone through sufficient usability testing with psychiatric population	40	4	6	15	4	5	4	2	0	0	3.5	1.6	0%	5%	33%	63%	3.0	4.0	3rd	Yes
HCP has doubts about the patient's ability to handle the required technology	40	13	12	10	1	3	0	1	0	0	2.3	1.4	0%	3%	10%	88%	1.9	2.8	3rd	Yes
HCP considers the device a hassle/too time consuming to appropriately bring in to care	40	18	11	6	2	2	1	0	0	0	2.1	1.3	0%	0%	13%	88%	1.6	2.5	3rd	Yes
Patient does not have access to the required technology	40	19	13	5	1	1	0	1	0	0	1.9	1.3	0%	3%	5%	93%	1.5	2.3	3rd	Yes

7c. Liability and logistical issues

	N	*"1"*	*"2"*	*"3"*	*"4"*	*"5"*	*"6"*	*"7"*	*"8"*	*"9"*	*Avg*	*SD*	*Chc*	*1st*	*2nd*	*3rd*	*CI_lo*	*CI_hi*	*Rank*	*Consensus*
Concern about patient confidentiality	40	3	7	16	7	1	2	1	3	0	3.5	1.8	0%	10%	25%	65%	3.0	4.1	3rd	Yes
Uncertainty about the frequency with which the digital data should be reviewed	40	4	7	10	8	6	4	1	0	0	3.5	1.6	0%	3%	45%	53%	3.0	4.0	3rd	Yes
Uncertainty about how to use the information clinically	40	7	8	8	8	8	0	1	0	0	3.2	1.5	0%	3%	40%	58%	2.7	3.6	3rd	Yes
Availability of too much information (e.g., HCP overwhelmed with additional patient information that needs consideration despite time-pressured schedule)	40	12	8	13	3	1	2	0	1	0	2.6	1.6	0%	3%	15%	83%	2.1	3.1	3rd	Yes
Time/disruption involved in integrating digital data into clinical practice	40	11	9	12	5	3	0	0	0	0	2.5	1.2	0%	0%	20%	80%	2.1	2.9	3rd	Yes
Difficulty having the digital health tool approved by insurance	40	14	11	8	3	3	0	0	1	0	2.4	1.5	0%	3%	15%	83%	1.9	2.8	3rd	Yes
Uncertainty about receiving reimbursement for time spent training or using the device and its data	40	14	11	8	3	3	1	0	0	0	2.3	1.4	0%	0%	18%	83%	1.9	2.7	3rd	Yes
Concern about potential for increased liability exposure due to not having effected or documented a clinical response to available information	39	15	10	9	3	1	1	0	0	0	2.2	1.3	0%	0%	13%	87%	1.8	2.6	3rd	Yes

7d. Unintended consequences

	N	*"1"*	*"2"*	*"3"*	*"4"*	*"5"*	*"6"*	*"7"*	*"8"*	*"9"*	*Avg*	*SD*	*Chc*	*1st*	*2nd*	*3rd*	*CI_lo*	*CI_hi*	*Rank*	*Consensus*
Patient discontinues needed traditional face-to-face treatment in favor of self-management	40	2	4	6	7	3	5	5	7	1	5.0	2.3	3%	33%	38%	30%	4.3	5.7	2nd	No
Distraction from therapy targets and tasks identified in session	40	3	2	6	7	7	6	7	2	0	4.7	1.9	0%	23%	50%	28%	4.1	5.3	2nd	No

Question 16. Training for Health Care Professionals (HCPs) In the next two questions, we ask you to rate a number of different types of training and training resources for HCPs. In 16a, we ask you to rate how important different types of training and training resources would be in helping health care professionals prescribe and interact with a digital health tool. In 16b, we ask how likely you believe HCPs would be to participate in different types of training. Assume the HCP will receive computerized reports with the output from the digital tool.

16a. Importance to HCP's ability to prescribe and interact with digital health tool

Please rate each item on a scale from 1 to 9 with 1 = not at all important 5 = somewhat important 9 = extremely important

	N	*"1"*	*"2"*	*"3"*	*"4"*	*"5"*	*"6"*	*"7"*	*"8"*	*"9"*	*Avg*	*SD*	*Chc*	*1st*	*2nd*	*3rd*	*CI_lo*	*CI_hi*	*Rank*	*Consensus*
Clear rationale provided to HCPs about how using this device can improve outcomes	39	0	0	0	0	0	2	7	9	21	8.3	0.9	54%	95%	5%	0%	8.0	8.6	Chc	Yes
Provision of hands-on work with device/dashboard during training sessions	39	0	0	0	0	1	4	10	6	18	7.9	1.2	46%	87%	13%	0%	7.6	8.3	1st	Yes
Inclusion of clinical examples and case materials as core elements of the training.	39	0	0	1	0	2	0	11	8	17	7.9	1.4	44%	92%	5%	3%	7.4	8.3	1st	Yes
Clear and concise tutorial provided in the digital device	39	0	0	0	1	3	2	8	12	13	7.7	1.3	33%	85%	15%	0%	7.3	8.1	1st	Yes
Technical call center support	39	0	0	0	0	3	1	13	14	8	7.6	1.1	21%	90%	10%	0%	7.2	7.9	1st	Yes
Prepared handouts to give to patients	38	0	0	1	2	3	1	12	7	12	7.4	1.6	32%	82%	16%	3%	6.9	7.9	1st	Yes
In-person training sessions	39	0	0	0	1	4	7	6	10	11	7.4	1.5	28%	69%	31%	0%	6.9	7.8	1st	Yes
Simple platform that can be learned with user guide and video demonstration without requiring in-person training	38	0	0	2	1	4	3	6	10	12	7.3	1.8	32%	74%	21%	5%	6.8	7.9	1st	Yes
Having the HCP use the digital system as a "patient" for a trial period to become familiar with its features	39	0	1	0	2	3	3	12	7	11	7.2	1.7	28%	77%	21%	3%	6.7	7.8	1st	Yes
Complete protocol and user guide	39	0	1	1	0	2	4	12	13	6	7.2	1.5	15%	79%	15%	5%	6.7	7.7	1st	Yes
Training provided in HCP's office (detailing approach)	39	0	0	2	0	5	7	2	14	9	7.2	1.7	23%	64%	31%	5%	6.7	7.7	1st	Yes
Availability of follow-up training sessions (if needed)	39	0	0	0	3	1	7	9	13	6	7.2	1.4	15%	72%	28%	0%	6.7	7.6	1st	Yes
CME credit for completing training	39	1	0	0	0	2	10	10	5	11	7.2	1.6	28%	67%	31%	3%	6.7	7.7	1st	Yes
In-person training followed by web-based video reinforcement	39	0	0	1	3	5	5	8	10	7	6.9	1.7	18%	64%	33%	3%	6.4	7.4	2nd	Yes
Video-based online training module	39	0	0	2	2	4	5	15	9	2	6.6	1.5	5%	67%	28%	5%	6.2	7.1	2nd	Yes
Training webinars	39	0	0	1	2	5	4	21	6	0	6.5	1.2	0%	69%	28%	3%	6.2	6.9	2nd	Yes
FAQs/user forums on a website	39	0	1	2	2	3	9	10	8	4	6.5	1.7	10%	56%	36%	8%	6.0	7.1	2nd	Yes
Online chat support for questions/technical issues	39	2	0	1	3	7	8	10	6	2	6.1	1.8	5%	46%	46%	8%	5.5	6.6	2nd	Yes
Work with medical schools to provide training in digital health technology	39	0	2	3	2	8	9	8	2	5	5.9	1.9	13%	38%	49%	13%	5.4	6.5	2nd	Yes
Availability of consultation groups (e.g., groups involving other HCPs)	38	0	2	3	6	6	7	8	3	3	5.7	1.9	8%	37%	50%	13%	5.1	6.3	2nd	Yes

16b. Likelihood that HCP would participate in this type of training

Please rate each item on a scale from 1 to 9 with 1 = not at all likely 5 = somewhat likely 9 = extremely likely

	N	*"1"*	*"2"*	*"3"*	*"4"*	*"5"*	*"6"*	*"7"*	*"8"*	*"9"*	*Avg*	*SD*	*Chc*	*1st*	*2nd*	*3rd*	*CI_lo*	*CI_hi*	*Rank*	*Consensus*
Clear and concise tutorial provided in the digital device	38	0	1	1	1	2	1	11	12	9	7.3	1.7	24%	84%	11%	5%	6.8	7.9	1st	Yes
Individualized training provided in HCP's office	38	0	0	2	1	2	7	9	6	11	7.2	1.7	29%	68%	26%	5%	6.6	7.7	1st	Yes
Technical call center support	38	0	2	0	6	2	7	10	7	4	6.4	1.9	11%	55%	39%	5%	5.8	7.0	2nd	Yes
Training webinars	38	0	1	1	3	8	7	14	4	0	6.0	1.4	0%	47%	47%	5%	5.6	6.5	2nd	Yes
Follow-up training sessions (if needed)	38	1	0	4	1	6	8	11	7	0	6.0	1.7	0%	47%	39%	13%	5.5	6.5	2nd	Yes
In-person training sessions	38	0	1	5	3	6	6	11	1	5	5.9	1.9	13%	45%	39%	16%	5.3	6.5	2nd	Yes
Having the HCP use the digital system as a "patient" for a trial period to become familiar with its features	38	2	4	1	2	2	9	10	4	4	5.9	2.3	11%	47%	34%	18%	5.1	6.6	2nd	No
Video-based online training module	38	0	1	3	3	9	8	8	4	2	5.8	1.7	5%	37%	53%	11%	5.3	6.4	2nd	Yes
Complete protocol and user guide	38	0	0	5	9	4	6	5	6	3	5.7	1.9	8%	37%	50%	13%	5.1	6.3	2nd	Yes
FAQs/user forums on a website	38	2	1	4	2	6	8	6	8	1	5.7	2.1	3%	39%	42%	18%	5.0	6.4	2nd	No
In-person training followed by web-based video reinforcement	38	0	2	4	5	5	9	8	5	0	5.6	1.8	0%	34%	50%	16%	5.0	6.1	2nd	Yes
Online chat support for questions/technical issues	38	1	2	6	5	5	8	6	4	1	5.2	2.0	3%	29%	47%	24%	4.6	5.9	2nd	No
Consultation groups (e.g., groups involving other HCPs)	38	3	3	8	1	4	8	10	1	0	4.8	2.1	0%	29%	34%	37%	4.1	5.5	2nd	No

Question 17. Provision of training and support to PATIENTS by HCPs Assume that the digital health tool designed for use with a patient with serious mental illness will be prescribed and monitored by an HCP. When we refer to HCPs here, we mean any professional providing health care services to patients with mental illnesses (e.g., psychiatrist, psychologist, nurse practitioner, clinical social worker). Thus, the HCP will be part of the system so that the design of the HCP's participation must be optimized. In the next two questions, we ask you to rate a number of different types of training and training resources for patients that would be provided by HCPs in terms of 17a) how important they would be in helping patients successfully engage with and use a digital health tool and then 17b) how difficult you believe each activity would be for the average HCP. Assume the HCP will receive computerized reports with the output from the digital tool.

17a. How IMPORTANT would each activity be in order to enable patients to successfully engage with and use a digital health tool?

Please rate each item on a scale from 1 to 9 with 1 = not at all important 5 = somewhat important 9 = extremely important

	N	*"1"*	*"2"*	*"3"*	*"4"*	*"5"*	*"6"*	*"7"*	*"8"*	*"9"*	*Avg*	*SD*	*Chc*	*1st*	*2nd*	*3rd*	*CI_lo*	*CI_hi*	*Rank*	*Consensus*
Initial training of patients on the system	39	0	0	0	0	0	1	6	7	25	8.4	0.9	64%	97%	3%	0%	8.2	8.7	Chc	Yes
Responding to technical problems identified by patients as quickly as possible	39	0	0	0	0	2	1	10	10	16	7.9	1.1	41%	92%	8%	0%	7.6	8.3	1st	Yes
Verbalizing or otherwise providing specific and clear feedback about the data gathered via the digital health tools during treatment sessions	39	0	0	0	0	1	3	11	14	10	7.7	1.0	26%	90%	10%	0%	7.4	8.1	1st	Yes
Providing reinforcement for patient's continued engagement with tools, independent of clinical information reported or outcomes within the first week of use	39	0	0	0	1	2	3	8	15	10	7.6	1.2	26%	85%	15%	0%	7.3	8.0	1st	Yes
Arranging for treatment coordinator or other member of the treatment team to provide ad hoc support for system use	39	0	0	0	0	1	4	14	13	7	7.5	1.0	18%	87%	13%	0%	7.2	7.9	1st	Yes
Developing ongoing reinforcement/reward systems for successful engagement with tools and/or successful self-management	38	0	0	1	0	2	3	8	17	7	7.5	1.3	18%	84%	13%	3%	7.1	7.9	1st	Yes
Helping patients set goals and tasks on digital health tools	39	0	0	0	1	2	4	12	9	11	7.5	1.3	28%	82%	18%	0%	7.1	7.9	1st	Yes
Identifying trends in digital data and responding immediately to critical clinical information	39	0	1	0	0	1	5	13	9	10	7.4	1.4	26%	82%	15%	3%	7.0	7.9	1st	Yes
Providing reinforcement for patient's continued engagement with tools, independent of clinical information reported or outcomes on an ongoing basis	39	0	0	0	1	3	3	13	10	9	7.4	1.3	23%	82%	18%	0%	7.0	7.8	1st	Yes
Follow-up/ongoing training on the system	39	0	0	0	2	3	7	12	7	8	7.1	1.4	21%	69%	31%	0%	6.7	7.5	1st	Yes
Initial training of family caregivers (if applicable) on the system	38	1	0	0	3	3	7	7	10	7	6.9	1.8	18%	63%	34%	3%	6.3	7.5	2nd	Yes
Verbalizing or otherwise providing specific and clear feedback about the data gathered via the digital health tools between treatment sessions	39	0	2	0	3	4	9	10	7	4	6.5	1.7	10%	54%	41%	5%	5.9	7.0	2nd	Yes
Sharing information with family/caregivers	38	1	0	2	4	13	9	3	6	0	5.6	1.6	0%	24%	68%	8%	5.1	6.1	2nd	Yes

17b. How DIFFICULT would each activity would be for the average HCP?

Please rate each item on a scale from 1 to 9 with 1 = extremely difficult 5 = somewhat difficult 9 = not at all difficult

	N	*"1"*	*"2"*	*"3"*	*"4"*	*"5"*	*"6"*	*"7"*	*"8"*	*"9"*	*Avg*	*SD*	*Chc*	*1st*	*2nd*	*3rd*	*CI_lo*	*CI_hi*	*Rank*	*Consensus*
Verbalizing or otherwise providing specific and clear feedback about the data gathered via the digital health tools during treatment sessions	39	0	0	4	4	5	4	13	7	2	6.2	1.7	5%	56%	33%	10%	5.7	6.7	2nd	Yes
Providing reinforcement for patient's continued engagement with tools, independent of clinical information reported or outcomes within the first week of use	39	0	1	2	4	7	5	12	5	3	6.2	1.7	8%	51%	41%	8%	5.6	6.7	2nd	Yes
Helping patients set goals and tasks on digital health tools	39	0	1	6	1	8	7	9	3	4	5.9	1.9	10%	41%	41%	18%	5.3	6.5	2nd	Yes
Initial training of patients on the system	39	0	1	6	7	11	4	5	2	3	5.3	1.8	8%	26%	56%	18%	4.7	5.8	2nd	Yes
Arranging for treatment coordinator or other member of the treatment team to provide ad hoc support for system use	39	0	4	7	4	5	7	7	3	2	5.2	2.1	5%	31%	41%	28%	4.6	5.8	2nd	No
Follow-up/ongoing training on the system	38	0	3	3	8	9	7	4	2	2	5.2	1.8	5%	21%	63%	16%	4.6	5.7	2nd	Yes
Providing reinforcement for patient's continued engagement with tools, independent of clinical information reported or outcomes on an ongoing basis	39	0	4	5	10	4	2	9	4	1	5.1	2.0	3%	36%	41%	23%	4.5	5.7	2nd	No
Developing ongoing reinforcement/reward systems for successful engagement with tools and/or successful self-management	39	0	5	4	5	11	7	4	2	1	4.9	1.8	3%	18%	59%	23%	4.4	5.5	2nd	Yes
Sharing information with family/caregivers	39	1	3	8	6	8	7	3	1	2	4.7	1.9	5%	15%	54%	31%	4.1	5.3	2nd	Yes
Initial training of family caregivers (if applicable) on the system	39	2	3	11	6	5	5	3	2	2	4.5	2.1	5%	18%	41%	41%	3.8	5.1	2nd	No
Identifying trends in digital data and responding immediately to critical clinical information	38	5	6	3	9	9	1	3	2	0	3.9	2.0	0%	13%	50%	37%	3.3	4.6	3rd	Yes
Verbalizing or otherwise providing specific and clear feedback about the data gathered via the digital health tools between treatment sessions	39	2	5	14	7	3	4	4	0	0	3.8	1.7	0%	10%	36%	54%	3.3	4.3	3rd	Yes
Responding to technical problems identified by patients as quickly as possible	39	8	7	10	5	3	3	2	1	0	3.3	1.9	0%	8%	28%	64%	2.7	3.9	3rd	Yes

Question 19. Patient characteristics that affect outcomes in psychotherapy/psychosocial interventions How much do you believe the following patient characteristics affect the chances of achieving favorable outcomes in psychotherapy/psychosocial interventions? Thinking about this question, please use your experience with all types of therapies/interventions and different types of patients.

Please rate each item on a scale from 1 to 9 with 1 = likely to have a very adverse effect on outcomes 5 = not likely to influence outcomes much one way or another 9 = extremely likely to promote good outcomes.

19a. Patient Characteristics

	N	*"1"*	*"2"*	*"3"*	*"4"*	*"5"*	*"6"*	*"7"*	*"8"*	*"9"*	*Avg*	*SD*	*Chc*	*1st*	*2nd*	*3rd*	*CI_lo*	*CI_hi*	*Rank*	*Consensus*
Positive expectations about therapy on the part of the patient	34	0	0	0	0	1	1	12	10	10	7.8	1.0	29%	94%	6%	0%	7.5	8.1	1st	Yes
Resources that facilitate access to treatment (availability of transportation, availability of HCPs trained to provide psychotherapy, insurance coverage)	34	0	0	0	0	0	3	11	13	7	7.7	0.9	21%	91%	9%	0%	7.4	8.0	1st	Yes
Positive social support (i.e., patient has an involved significant other or caregiver)	34	0	1	0	0	0	5	9	12	7	7.5	1.4	21%	82%	15%	3%	7.0	7.9	1st	Yes
Bachelor's degree or more education	34	0	0	0	0	7	12	11	4	0	6.4	0.9	0%	44%	56%	0%	6.0	6.7	2nd	Yes
Currently married	33	0	0	0	0	16	6	9	1	1	5.9	1.1	3%	33%	67%	0%	5.6	6.3	2nd	Yes
18-44 years old	34	1	0	1	0	16	6	6	3	1	5.7	1.5	3%	29%	65%	6%	5.2	6.2	2nd	Yes
< 18 years old	34	0	0	3	3	15	4	4	4	1	5.6	1.5	3%	26%	65%	9%	5.0	6.1	2nd	Yes
High school or more education	34	0	0	0	1	17	13	3	0	0	5.5	0.7	0%	9%	91%	0%	5.3	5.8	2nd	Yes
45-64 years old	34	0	0	0	5	16	7	3	3	0	5.5	1.1	0%	18%	82%	0%	5.1	5.9	2nd	Yes
Female gender	34	0	1	0	0	18	11	4	0	0	5.5	0.9	0%	12%	85%	3%	5.2	5.8	2nd	Yes
65+ years old	33	0	1	2	3	16	7	2	2	0	5.2	1.3	0%	12%	79%	9%	4.8	5.6	2nd	Yes
Male gender	34	0	1	0	7	24	1	1	0	0	4.8	0.8	0%	3%	94%	3%	4.5	5.1	2nd	Yes
Minority status	34	0	0	1	10	21	1	1	0	0	4.7	0.7	0%	3%	94%	3%	4.5	5.0	2nd	Yes
Low socioeconomic status	34	1	0	6	16	10	1	0	0	0	4.1	0.9	0%	0%	79%	21%	3.8	4.4	2nd	Yes
Did not complete high school	34	0	0	9	16	7	2	0	0	0	4.1	0.9	0%	0%	74%	26%	3.8	4.3	2nd	Yes
Low health literacy	34	0	4	12	11	7	0	0	0	0	3.6	1.0	0%	0%	53%	47%	3.3	3.9	3rd	Yes
Low literacy (i.e., reading ability)/low numeracy	34	0	4	15	10	5	0	0	0	0	3.5	0.9	0%	0%	44%	56%	3.2	3.8	3rd	Yes
Low IQ	34	1	5	14	9	4	1	0	0	0	3.4	1.1	0%	0%	41%	59%	3.0	3.7	3rd	Yes
Severe psychosocial stressors (e.g., poverty, general medical problems, abusive relationship, legal problems)	34	5	11	12	4	1	1	0	0	0	2.6	1.2	0%	0%	18%	82%	2.3	3.0	3rd	Yes
Serious level of chaos and disorganization in the person's life/environment	33	6	15	10	1	0	1	0	0	0	2.3	1.0	0%	0%	6%	94%	2.0	2.6	3rd	Yes
Low motivation	33	11	13	7	2	0	0	0	0	0	2.0	0.9	0%	0%	6%	94%	1.7	2.3	3rd	Yes

19b. Disorders, Signs, and Symptoms

	N	*"1"*	*"2"*	*"3"*	*"4"*	*"5"*	*"6"*	*"7"*	*"8"*	*"9"*	*Avg*	*SD*	*Chc*	*1st*	*2nd*	*3rd*	*CI_lo*	*CI_hi*	*Rank*	*Consensus*
Good occupational functioning	34	0	0	0	0	1	7	15	11	0	7.1	0.8	0%	76%	24%	0%	6.8	7.3	1st	Yes
Diagnosis of major depressive disorder	34	0	0	0	5	18	6	4	1	0	5.4	1.0	0%	15%	85%	0%	5.0	5.7	2nd	Yes
Diagnosis of bipolar disorder	34	0	2	1	6	18	4	3	0	0	4.9	1.1	0%	9%	82%	9%	4.5	5.3	2nd	Yes
Suicidal ideation	34	2	2	1	5	20	2	2	0	0	4.6	1.4	0%	6%	79%	15%	4.1	5.0	2nd	Yes
Diagnosis of schizophrenia	34	1	1	8	7	14	2	1	0	0	4.2	1.2	0%	3%	68%	29%	3.8	4.6	2nd	Yes
Sensitivity to overstimulation	34	0	2	6	10	15	0	1	0	0	4.2	1.0	0%	3%	74%	24%	3.9	4.6	2nd	Yes
Longer duration of illness	34	0	4	8	5	14	3	0	0	0	4.1	1.2	0%	0%	65%	35%	3.7	4.5	2nd	Yes
Non-suicidal self-injurious behaviors	34	1	4	7	4	15	2	1	0	0	4.1	1.4	0%	3%	62%	35%	3.7	4.6	2nd	Yes
Multiple previous episodes	33	3	1	5	9	13	1	1	0	0	4.1	1.4	0%	3%	70%	27%	3.6	4.5	2nd	Yes
Multiple comorbid Axis I disorders	34	2	2	8	11	10	0	1	0	0	3.9	1.3	0%	3%	62%	35%	3.4	4.3	3rd	Yes
Low frustration tolerance	34	0	2	13	15	3	1	0	0	0	3.6	0.8	0%	0%	56%	44%	3.4	3.9	3rd	Yes
Low energy/fatigue	34	0	2	16	11	5	0	0	0	0	3.6	0.8	0%	0%	47%	53%	3.3	3.8	3rd	Yes
Presence of Axis II comorbidity	34	3	2	12	11	5	1	0	0	0	3.5	1.2	0%	0%	50%	50%	3.1	3.9	3rd	Yes
Greater severity of delusions/paranoia	34	3	3	13	7	7	1	0	0	0	3.4	1.3	0%	0%	44%	56%	3.0	3.9	3rd	Yes
Aggression/agitation	34	1	6	12	12	2	1	0	0	0	3.3	1.0	0%	0%	44%	56%	3.0	3.7	3rd	Yes
Greater severity of neurocognitive impairment	34	0	12	8	9	5	0	0	0	0	3.2	1.1	0%	0%	41%	59%	2.8	3.6	3rd	Yes
Greater severity of negative symptoms	34	3	8	15	3	4	0	1	0	0	3.0	1.3	0%	3%	21%	76%	2.6	3.5	3rd	Yes
Greater severity of disorganization symptoms	34	3	7	17	4	2	0	1	0	0	3.0	1.2	0%	3%	18%	79%	2.6	3.4	3rd	Yes
Acute substance use	34	6	13	10	4	1	0	0	0	0	2.4	1.0	0%	0%	15%	85%	2.1	2.8	3rd	Yes

19c. Appraisals and Patient Experience

	N	*"1"*	*"2"*	*"3"*	*"4"*	*"5"*	*"6"*	*"7"*	*"8"*	*"9"*	*Avg*	*SD*	*Chc*	*1st*	*2nd*	*3rd*	*CI_lo*	*CI_hi*	*Rank*	*Consensus*
Positive bond with healthcare professional (good therapeutic alliance)	34	0	0	0	0	0	0	10	13	11	8.0	0.8	32%	100%	0%	0%	7.8	8.3	1st	Yes
Perception of treatment as beneficial	34	0	0	0	0	0	1	9	14	10	8.0	0.8	29%	97%	3%	0%	7.7	8.3	1st	Yes
Self-efficacy beliefs about ability to participate in and benefit from psychotherapy/psychosocial intervention	34	0	0	0	1	0	0	10	11	12	7.9	1.1	35%	97%	3%	0%	7.6	8.3	1st	Yes
Agreement with tasks and goals of treatment	34	0	0	0	0	0	1	13	10	10	7.9	0.9	29%	97%	3%	0%	7.6	8.2	1st	Yes
Willingness to complete tasks/homework between treatment sessions	34	0	0	0	1	1	4	9	8	11	7.6	1.3	32%	82%	18%	0%	7.2	8.1	1st	Yes
Readiness to change	34	0	0	0	1	1	6	5	12	9	7.6	1.3	26%	76%	24%	0%	7.1	8.0	1st	Yes
History of good adherence to medication and other treatment	34	0	0	0	2	0	3	15	9	5	7.3	1.2	15%	85%	15%	0%	6.9	7.7	1st	Yes
Psychological mindedness	34	0	0	0	0	4	9	11	6	4	6.9	1.2	12%	62%	38%	0%	6.5	7.3	1st	Yes
Engagement with mutual help organizations and/or peer support groups	34	0	0	1	1	6	10	7	6	3	6.5	1.4	9%	47%	50%	3%	6.0	7.0	2nd	Yes
Stigma/discomfort/social anxiety about seeking care for mental or behavioral problems	34	0	4	17	5	5	3	0	0	0	3.6	1.2	0%	0%	38%	62%	3.2	4.0	3rd	Yes
Negative past experience with psychotherapy/psychosocial intervention	33	1	4	17	7	3	1	0	0	0	3.3	1.0	0%	0%	33%	67%	3.0	3.6	3rd	Yes
Limited insight into disorder	34	5	8	10	8	2	1	0	0	0	2.9	1.3	0%	0%	32%	68%	2.5	3.3	3rd	Yes
